# Do probiotics modulate dietary intake? Pilot data from a randomized controlled sub-study of the ProBioHRV clinical trial in patients with depression and healthy controls

**DOI:** 10.1371/journal.pone.0350801

**Published:** 2026-06-23

**Authors:** Julia Corinna Putz, Marilena Wilding, Sonja Lackner, Martin Narrath, Daria Schlotmann, Marie-Therese Sallmutter, Jasmin Tatzer, Andreas Brandstätter, Julia Deborah Lang, Sandra Holasek, Julian Wenninger, Mary I. Butler, Susanne Bengesser, Lena Gruber, Andreas Baranyi, Jolana Wagner-Skacel, Sabrina Mörkl

**Affiliations:** 1 Department of Psychiatry, Psychosomatics and Psychotherapeutic Medicine, Division of Medical Psychology, Psychosomatics and Psychotherapy, Medical University of Graz, Graz, Styria, Austria; 2 Division of Immunology, Otto Loewi Research Center, Medical University of Graz, Graz, Styria, Austria; 3 Department of Psychiatry and Neurobehavioral Science, University College Cork, Cork, Munster, Ireland; 4 Department of Psychiatry, Psychosomatics and Psychotherapeutic Medicine, Division of Psychiatry and Psychotherapeutic Medicine in Graz, Graz, Styria, Austria; Hong Kong Baptist University, HONG KONG

## Abstract

**Background:**

The gut microbiome plays a central role in human health and is strongly influenced by diet. Probiotics can beneficially modulate the microbiome and, through the gut–brain axis, may affect mood, appetite, and food preferences. This randomized controlled trial examined whether three months of probiotic supplementation could alter dietary intake in individuals with major depression (*MD*) and healthy controls (*HC*).

**Methods:**

In this double-blind, placebo-controlled trial, 53 participants (23 with *MD*, 30 *HC*) received either a multi-strain probiotic or placebo twice daily for three months. Dietary intake was assessed at baseline and three follow-ups using the Vienna Food Record (VFR). Nutritional data were analyzed with nut.s® software and evaluated using mixed ANOVAs for repeated measures. These analyses represent additional data collected within the framework of the ProBioHRV study.

**Results:**

Changes across dietary measures were generally small, with only a limited number reaching statistical significance. Significant three-way interactions (*time × intervention × diagnosis*) emerged for vitamin D intake, dietary variety, folic acid, and diversity. In *HC*, probiotic supplementation was associated with higher vitamin D intake after one week, while in *MD*, a similar increase was observed after three months (trend level, *p* = .058). Conversely, participants receiving probiotics showed lower dietary variety and diversity scores at several time points. Across all time points, folic acid intake was lower in *MD* compared to *HC*, independent of intervention.

**Conclusion:**

Probiotic supplementation did not produce consistent changes in nutrient intake but showed exploratory, time- and group-dependent patterns for selected measures, including vitamin D intake and dietary variety and diversity. Given the pilot nature of the study, these findings are descriptive and hypothesis- generating. Larger, well-powered studies with objective nutritional and microbiome measures are required.

## Introduction

### Gut microbiome and dietary intake

The human gut harbors trillions of microorganisms, including prokaryotes, archaea, and eukaryotes, far exceeding the number of humans who have ever lived [1–3]. The intestinal microbiota plays a key role in energy extraction, vitamin synthesis (e.g., vitamin B12), bile acid metabolism, and dietary fiber breakdown via fermentation, hydrolysis, and deconjugation [4–6]. Microbial composition is dynamic and influenced by factors like age, sex, medications, environmental exposures, geographical origin, diseases, and notably diet [7–10]. Diets low in fiber and high in processed foods, sugars, fats, and animal proteins – typical of the Western diet – have been found to reduce gut microbial diversity [11,12] whereas fiber-rich diets like the Mediterranean diet seem to promote microbial diversity and anti-inflammatory stability [13,14].

The gut microbiome is highly responsive to dietary changes, with shifts observed after only one day on a high-fat diet in mice [15]. In humans, Western diets decrease beneficial bacteria like *Bifidobacteria* and *Eubacteria* [12,16,17], while Mediterranean diets enrich beneficial microbial populations and potentially counteract obesity and inflammation [13,14,18].

Alterations in gut microbiota composition are associated with obesity, often reflected in changes in the *Firmicutes/Bacteroidetes* ratio [19–21]. Some studies found a lower microbial diversity in obese individuals [19,22], although findings are not entirely consistent [19,23]. Similarly, dysbiosis has been linked to mental health conditions such as depression, bipolar disorder, autism spectrum disorder, anorexia nervosa and ADHD [24–27]. However, causal relationships remain to be fully clarified [28].

### Microbiome and appetite regulation

Gut bacteria may influence appetite regulation through interactions with hormonal and metabolic pathways. Key hormones include leptin, ghrelin, and insulin, alongside microbiota-derived metabolites like short-chain fatty acids (SCFA) [29].

Leptin, secreted mainly by adipose tissue, suppresses appetite via hypothalamic signaling [30–38]. Higher gut microbial diversity is associated with better leptin signaling, while lower diversity correlates with elevated leptin levels [29,39].

Conversely, ghrelin, the “hunger hormone” stimulates appetite and energy intake [40–43], while insulin promotes satiety and regulates appetite through hypothalamic pathways [44–47]. Notably, lower gut microbiome diversity has been linked to insulin resistance [39] and alterations in energy homeostasis [46,48].

Microbial influences on food preferences and eating patterns may involve modulation of taste receptors, neurotransmitter production, hormonal pathways, and the induction of dysphoria to favor specific nutrient intakes [49]. These mechanisms highlight a potential role of the gut microbiome in appetite regulation and food choice.

### Probiotics, diet and depression

Probiotics – live microorganisms offering health benefits – mainly include *Lactobacilli* and *Bifidobacteria* [50]. They are established treatments for gastrointestinal [51–53] and are increasingly studied for psychiatric disorders like depression and ADHD [27,52,54–59]. Further, probiotics have been suggested as an add-on therapy for depression in recent guidelines [60].

Importantly, probiotic intake has been associated with reductions in body weight, BMI, and appetite-related hormones such as ghrelin [61–68]. Furthermore, given the broad impact of probiotics via the gut-brain axis – such as modulation of HRV [69] and the reduction of depressive symptoms in patients with unipolar depression [54,55,57] – it would be valuable to investigate whether dietary changes also play a role in conjunction with probiotic intake. This is particularly relevant given that dietary alterations can influence the gut microbiome, and microbiome dysbiosis has been associated with psychiatric disorders such as depression [24–27]. Additionally, changes in dietary patterns have been associated with improvements in patients with depression [70]. This relationship is a central focus of the emerging field of “nutritional psychiatry,” which examines how macro- and micronutrients influence neurotransmitter synthesis and, in turn, affect the development and course of psychiatric disorders [58,70,71].

### Research question and hypotheses

Building on these findings, we aimed to investigate whether daily probiotic supplementation over a period of three month influences dietary intake (i.e., the consumption of macro- and micronutrients) in individuals with major depression (*MD*) and healthy controls (*HC*).

We hypothesized that probiotic intervention would change patterns of dietary intake such as variety and diversity by modulating appetite-regulating pathways, potentially fostering healthier eating patterns and improved nutritional quality, which may contribute to both physiological well-being and mental health outcomes.

## Materials and methods

### Participants

The details of the study design have already been described in Mörkl et al. (2025) and will therefore only be briefly summarized here [69].

**Fig 1:**
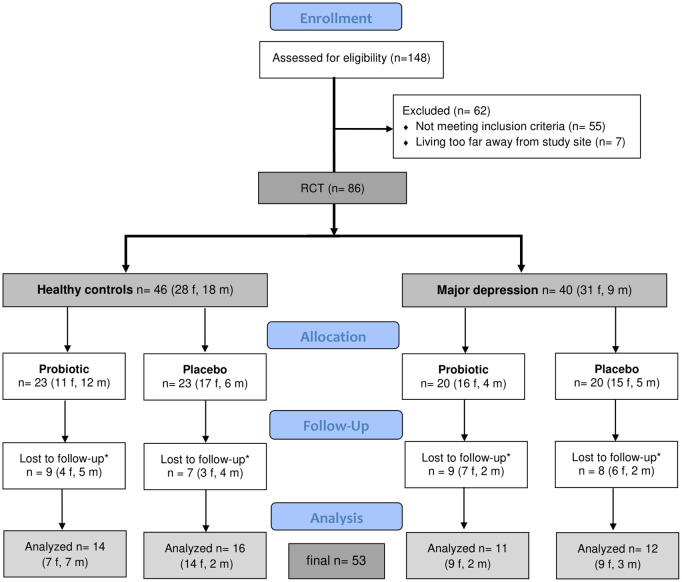
CONSORT flow chart illustrating the recruitment process and associated drop-outs. *Drop-out was due to incomplete dietary assessments.

All data were collected between 16 August 2021 and 27 June 2023 at the University Clinic for Psychiatry and Psychotherapeutic Medicine in Graz as part of the project *“Probiotics and the gut-brain axis: Do probiotics interact with the vagus nerve?”* Participants were recruited at the Department for Psychiatry and Psychotherapeutic Medicine, on social media or via the online recruitment-service Probando (www.probando.io), resulting in 86 volunteers. Due to incomplete food frequency questionnaire data, 33 participants had to be excluded from the statistical analysis, resulting in 53 participants between 18 and 67 years (see Fig 1). Participants had either a diagnosis of major depression (*n* = 23; depression group, *MD*) or were free of psychiatric diseases (*n* = 30; healthy controls, *HC*). Patients with *MD* and *HC* were each randomly assigned to either a *probiotic* (*n* = 25) or *placebo* (*n* = 28) group, resulting in four condition groups. The final group sizes for analysis differ slightly due to drop-outs during the intervention period (see Table 1).

**Table 1:** Anthropometric and questionnaire data for all groups.

	*Placebo (n = 28)*	*Probiotics (n = 25)*	*p*	*HC (n = 30)*	*MD (n = 23)*	*p*	*MD Probiotics (n = 11)*	*HC Probiotics (n = 14)*	*p*	*MD Placebo (n = 12)*	*HC Placebo (n = 16)*	*p*
***Sex (female)***	23	16	*.212*	21	18	*.547*	9 (2)	7 (7)	*.208*	9 (3)	14 (2)	*.624*
***Smoking status***	4	6	*.488*	4	6	*.300*	3	3	*1.000*	3	1	*.285*
***Age (years)***	35.0 (25.0-53.5)	36.0 (24.0-44.5)	*.475*	34.5 (25.0 −46.8)	36.0 (24.0-52.0)	*.767*	31.0 (22.0-39.0)	36.00 (26.50-49.50)	*.159*	44.50 (25.00-56.50)	31.00 (25.00-45.75)	*.450*
***Weight (kg)***	66.3 (59.5-75.1)	64.0 (58.6-84.0)	*.708*	62.4 (56.7-76.1)	68.3 (60.7-82.5)	*.209*	70.0 (60.7- 93.0)	60.45 (56.23-75.38)	*.183*	67.80 (59,65-73.03)	64.15 (57.65-77.03)	*.732*
***Height (cm)***	169.54 (9.07)	170.88 (7.96)	*.571*	170.37 (7.69)	169.91 (9.64)	*.850*	170.18 (7.51)	171.43 (8.53)	*.706*	169.67 (11.60)	169.44 (7.01)	*.949*
***BMI (kg/m²)***	22.2 (20.4-26.1)	22.9 (20.9 −27.2)	*.510*	21.8 (20.7-25.6)	23.6 (21.0-27.0)	*.151*	23.8 (21.1-30.6)	22.05 (20.78-25.64)	*.109*	22.96 (20.67-26.06)	21.55 (20.02-25.95)	*.537*
***BDI (t1)***	11.68 (12.34)	11.88 (12.34)	*.818*	4.34 (4.13)	21.13 (12.65)	<.001***	19.73 (13.99)	5.23 (4.95)	.007**	22.42 (11.77)	3.62 (3.30)	<.001***
***HAMD (t1)***	8.75 (10.73)	10.29 (12.01)	*.342*	1.83 (1.34)	19.09 (10.85)	<.001***	19.91 (11.91)	2.15 (1.34)	<.001***	18.33 (10.25)	1.56 (1.31)	<.001***

Note. Values are presented as mean (SD); non-normally distributed variables are reported as median (IQR). P-values indicate differences between the two adjacent groups to the left. Statistical significance was set at p < .05. **p < .01; ***p < .001. Significant results are shown in bold.

All participants underwent a structured clinical interview (M.I.N.I.-International Neuropsychiatric Interview) conducted by a trained psychiatrist or psychologist to either confirm the *MD* diagnosis or to exclude *HC* with any psychiatric diagnosis, according to the International Classification of Diseases (ICD-10) [72]. Further exclusion criteria for all participants were: suicidal tendencies, lack of consent or capacity to consent, known cardiovascular diseases, pregnancy, breastfeeding, pronounced dependence on alcohol or psychotropic substances (benzodiazepines, morphines), other severe mental or organic diseases (epilepsy, brain tumors, trauma, severe recent surgery), tumor diseases, dementia (Mini Mental Score <20), severe autoimmune diseases or immunosuppression (lupus erythematosus, HIV, multiple sclerosis), antibiotic therapy in the last month, laxative abuse, acute infections, diarrhea and gastrointestinal surgery (except appendectomy). Participants were required to not have taken probiotics in the preceding 6 months. Furthermore, they were instructed to refrain from consuming any additional probiotics during the study period. In addition, the intake of antibiotics and prebiotic supplements was an exclusion criterion. All participants provided written informed consent. Each participant was assigned an individual number, and the data were pseudonymized to prevent any identification by name. Identification of participants was possible solely by the authors, and only if required.

### Study design

Participants had to visit the clinic at four different time points for data collection: at baseline (before the intervention; *t1*), 7 days after intervention start (*t2*), after 28 days (*t3*), and after 3 months (end of intervention; *t4*). These visits included the collection of a blood sample, saliva and stool samples, a clinical interview, a 24-hour heart rate variability measurement, and several questionnaires assessing psychological and cognitive measures, sleep quality as well as dietary intake measured by a food frequency questionnaire, the Vienna Food Record (VFR; see 2.4). In this study, we only analyzed dietary intake using the VFR.

The study was registered on clinicaltrials.gov (NCT-04772664) on August 16, 2021, and received ethical approval (EK-No: 33–227 ex 20/21). All participants provided written informed consent.

### Probiotic intervention

Participants in the probiotic group received a multispecies probiotic (*OMNi-BiOTiC® STRESS Repair*) from Allergosan® (Graz, Austria) for a period of three months. They consumed one sachet (3g) twice daily, providing a total of 1.5 × 10¹⁰ CFU. This probiotic contained nine bacterial species: *Bifidobacterium bifidum* W23, *Bifidobacterium lactis* W51 and W52, *Lactobacillus acidophilus* W22, *Lactobacillus casei* W56, *Lactobacillus paracasei* W20, *Lactobacillus plantarum* W62, *Lactobacillus salivarius* W24, and *Lactococcus lactis* W19 with at least 7.5 billion microorganisms per sachet. These species were selected for their anti-inflammatory properties [73], their ability to reduce cognitive reactivity to sad mood [74] and their potential to activate the vagus nerve, as demonstrated in animal models [75–78].

The placebo group received identical-looking sachets in color, consistency, and taste, containing maize starch, maltodextrin, inulin, potassium chloride, magnesium sulfate, fructooligosaccharides, amylases and manganese sulfate. The probiotic and placebo powders had to be mixed with water and consumed twice daily (morning and evening).

The study followed a double-blind design. Participants were instructed to adhere to the intervention, refrain from taking additional probiotics, and maintain their usual diet, lifestyle and physical activity. Patients with *MD* continued their standard psychopharmacological treatment and usual care.

### Questionnaires

General clinical and demographic data of participants (age, weight, height, BMI, sex, smoking, medication) were assessed. At all four time points, standardized questionnaires were administered.

The Vienna Food Record (VFR) was administered at all four study visits to document the participants’ dietary intake within the past 24 hours. The VFR is a validated, paper-based, pre-coded dietary record specifically developed for Austrian adults, comprising 182 predefined food items representative of typical national dietary habits. It includes a brief instruction page and portion size illustrations (e.g., standard weights for common foods such as bread, meat or beverages), allowing participants to estimate quantities without professional assistance. Food consumption is recorded in predefined categories, including main meals, snacks and beverages with the option to note preparation details and consumption outside the home. [79]. In our study, a 24-h dietary record was evaluated at each visit. This approach was chosen to minimize participant burden and to account for the limited cognitive performance and resilience often present in *MD patients*. Given the additional completion of questionnaires within the study protocol, a higher workload was expected to markedly reduce compliance. Restricting the assessment to one day per visit was therefore considered the most feasible approach to ensure reliable data collection.

Other questionnaires included the Beck Depression Inventory (BDI) [80], which is a standardized instrument for self-assessment of the severity of depressive symptoms and the Hamilton Scale (HAMD) [81] – a clinical scale for evaluating depression severity. These questionnaires were administered to assess symptom-changes over time; all patients were included in the study regardless of depression severity (mild, moderate, or severe).

Furthermore, participants were asked to retrospectively assess their adherence to the daily probiotic/placebo intake over the three-month period using a questionnaire, in which they could choose from five predefined response categories: 0 = Not at all, 1 = Rarely, 2 = Mostly, 3 = Almost always, and 4 = Always.

### Data analysis

To analyze the data obtained from the Vienna Food Record, the nutritional software nut.s® (v1 33.20, dato.denkwerkzeuge, Vienna) was used [82,83]. The software allows to calculate the nutritional values of different foods and recipes and to evaluate a person’s nutritional status. Its database is based on the German Food Composition Database (Bundeslebensmittelschlüssel, BLS) and the Austrian Food Composition Database (Österreichische Nährwerttabelle, ÖNWT). For further analysis, the following dietary intake parameters were selected: carbohydrates [g], proteins [g], total fats [g], energy [kcal], omega-3 fatty acids [mg], omega-6 fatty acids [mg], saturated fatty acids [mg], vitamin B6 [mg], vitamin B12 [μg], vitamin D [µg], iron [mg], magnesium [mg], folic acid [mg], and dietary fiber [g]. These nutrients were chosen due to their relevance for neurotransmitter synthesis, general health, microbiome interactions and their specific role in psychiatric disorders, particularly depression [84].

Additionally, dietary variety (the number of different individual foods consumed) and dietary diversity (the number of different food groups represented in the diet) were assessed.

Notably, this analysis did not take into account the intake of any food supplements reported in the dietary protocol.

### Statistical analysis

For the statistical analysis, IBM SPSS Statistics for Windows (Version 29.0. Armonk, NY: IBM Corp.) was used.

#### Sample size, randomization, and blinding.

This analysis was conducted within the framework of the *ProBioHRV* trial, which investigated the effects of a multi-species probiotic (OMNi-BiOTiC® STRESS Repair) on vagus nerve function, gut microbiota composition, and related clinical outcomes in patients with major depression (MD) and healthy controls (HC). The original trial included 86 participants (43 MD, 43 HC). For the present study, dietary data were available from 53 participants (23 MD, 30 HC), who were therefore included in this sub-analysis.

#### Sample size determination.

The planned total sample size of 80 participants (20 per group; HC: probiotic/placebo and MD: probiotic/placebo) was calculated using the Repeated Measures ANOVA (within–between interaction) procedure in G*Power (version 3.1).

Assumptions included a small effect size (f = 0.2), alpha = 0.05, and power = 0.95, ensuring adequate sensitivity to detect time × group × intervention interactions. Recruitment yielded 86 participants, exceeding the calculated requirement; however, only those with complete dietary data (n = 53) were analyzed here.

#### Randomization and allocation concealment.

Randomization was performed by the manufacturer of the probiotic and placebo using block randomization via www.randomization.com. The random allocation sequence was generated by a single staff member at the manufacturing site who was not otherwise involved in the study. Randomization was performed separately within each diagnostic group. The initial sample comprised 86 participants (43 with MD and 43 healthy controls), who were randomly assigned within group to either the probiotic or placebo intervention. This resulted in 20 participants with MD and 23 healthy controls for each intervention group. Reported subgroup sizes reflect the final sample after participant drop-out due to incomplete dietary assessment (see Table 1).

To maintain allocation concealment, the manufacturer prepared sequentially numbered, opaque, sealed packages according to the randomization list. Each participant received three packages, one for each month of treatment, labeled solely with the participant identification code. The probiotic and placebo products were identical in appearance, color, weight, smell, and taste, ensuring indistinguishability.

#### Blinding.

Both the person responsible for randomization and all unblinded manufacturer personnel were sworn to confidentiality. All other members of the study team – including investigators, clinicians, participants, assessors, and data analysts – remained fully blinded until the database was locked after study completion.

#### Handling of missing data.

No imputation was performed for missing data, as complete dietary and clinical datasets were available for all 53 participants included in the present analysis.

#### Descriptive statistics.

Descriptive statistics were calculated to summarize baseline characteristics, including means and standard deviations for continuous variables, as well as absolute and relative frequencies for categorical variables.

#### Inferential statistics.

To assess the comparability of the groups at baseline, all demographic and clinical variables were subjected to appropriate statistical testing. Categorical variables such as sex and smoking status were analyzed using Chi-square tests; in cases of low expected cell counts, the Fisher’s exact test was applied. Continuous variables, including age, height, weight, BMI, as well as the psychometric scores BDI and HAMD, were first tested for normality using the Shapiro–Wilk test. Depending on the distribution, either independent samples t-tests (for normally distributed data) or Mann–Whitney U tests (for non-normally distributed data) were used to compare the groups. This approach ensured that baseline differences were appropriately accounted for in the subsequent analyses.

Potential differences in participant adherence between intervention and diagnosis groups were compared using Mann-Whitney-U tests.

To analyze changes in the participants’ dietary nutrient intake for each parameter, mixed analyses of variance (ANOVA) for repeated measures were used. The analysis included the within-subjects factor *time*, corresponding to the four measurement time points (*t1- t4*), and the between-subjects factors *intervention* (*placebo* vs. *probiotic group*) and *diagnosis* (*MD, HC*). In addition, post hoc analyses were performed to elucidate group differences. Post-hoc comparisons within each ANOVA were Bonferroni-corrected; however, we did not adjust for the number of ANOVAs, as corrections across conceptually distinct analyses are not recommended since they may inflate Type II error [85].

To verify that the assumptions required for conducting ANOVA were met, we assessed data sphericity using Mauchly’s test, and homogeneity of variances using Levene’s test.

Normality was assessed by examining the unstandardized residuals using the Shapiro–Wilk test. Data for carbohydrates [g], proteins [g], total fats [g], energy [kcal], and saturated fatty acids [mg] followed a normal distribution (all *p* > .05). Variables that showed substantial deviations from normality, defined as significant Shapiro–Wilk results (*p* < .05) in at least 50% of the groups, were log-transformed to meet the assumptions of parametric testing (they all showed a right-skewed distribution). These included vitamin D [µg], folic acid [μg], fiber [g], variety, omega-3 fatty acids [mg], omega-6 fatty acids [mg], vitamin B6 [mg], vitamin B12 [μg], and iron [mg]. After log-transformation, Shapiro Wilk tests confirmed normal distribution of the data (all *p* > .05).

To rule out a potential attrition bias, we further conducted a drop-out analysis, comparing included individuals and individuals excluded due to incomplete dietary records in demographic parameters and depressive symptom severity at baseline (height, weight, BMI, depressive symptoms measured by HAMD, depressive symptoms measured by BDI, age, smoking status, diagnosis, and distribution of sex). We tested group differences using independent samples t-tests and Mann- Whitney-U tests and Chi square tests, depending on scale level and variable distribution.

## Results

### Descriptive data

The total sample comprised 53 participants, including 28 individuals in the placebo group and 25 in the probiotic group. Of these, 30 were *HC* and 23 had been diagnosed with *MD*. The mean age of participants was 36.68 years (SD = 13.77), and the sample included 39 women (73.6%) and 14 men (26.4%).

### Group differences

Inferential analyses revealed no significant differences in sex, age, height, weight, or smoking status between the *probiotic* and *placebo* groups, between participants with *MD* and *HC* and between the *probiotic* and *placebo* groups when comparing *MD* and *HC*. As expected, baseline comparisons showed significant differences in BDI and HAMD scores between *MD* and *HC* participants – both overall and within the *probiotic* and *placebo* subgroups. However, no significant differences in these psychological measures were observed between the *probiotic* and *placebo* groups. Table 1 provides a summary of these results.

### Adherence

Mann-Whitney-U tests revealed a significant difference in subjective adherence in the *intervention* group (Z = −2.28, *p* = .024), with individuals taking probiotics showing higher adherence. There was no significant difference in the *diagnosis* group in their subjective adherence (Z = −1.50, *p* = .142).

### ANOVA results

There were no significant violations of sphericity or homogeneity of variances between our groups, as tested using Mauchly’s test and Levene’s test in most nutrients, respectively (all *p* > .05). For vitamin D, sphericity was violated and therefore the Greenhouse-Geisser corrected values are presented.

After log-transformation, data did not differ significantly from a normal distribution (all *p* > .05).

As the results obtained from log-transformed and untransformed data were highly comparable, we report the results based on the untransformed data for greater clarity and ease of interpretation.

Baseline comparability between groups (*placebo* vs. *probiotic*; *MD* vs. *HC*) was assessed using post-hoc pairwise comparisons of respective groups at *t1.* No significant differences were observed between any of the groups at *t1* with respect to vitamin D, fiber intake, and dietary diversity (all *p* > .05). However, participants in *MD* and *HC* differed significantly in folic acid intake at *t1*, with *HC* showing higher values than *MD* (*p* = .029). Moreover, intervention groups differed in their dietary variety at *t1* (*p* = .034), with *placebo* group showing a significantly higher variety than *probiotics* group.

#### Main results.

We found no significant differences between groups (*intervention*, *diagnosis*) and time points in the following nutrients: carbohydrates [g], proteins [g], total fats [g], energy [kcal], saturated fatty acids [mg], omega-3 fatty acids [mg], omega-6 fatty acids [mg], vitamin B6 [mg], vitamin B12 [μg], iron [mg], and magnesium [mg], all *p* > .05.

#### Vitamin D.

**Fig 2:**
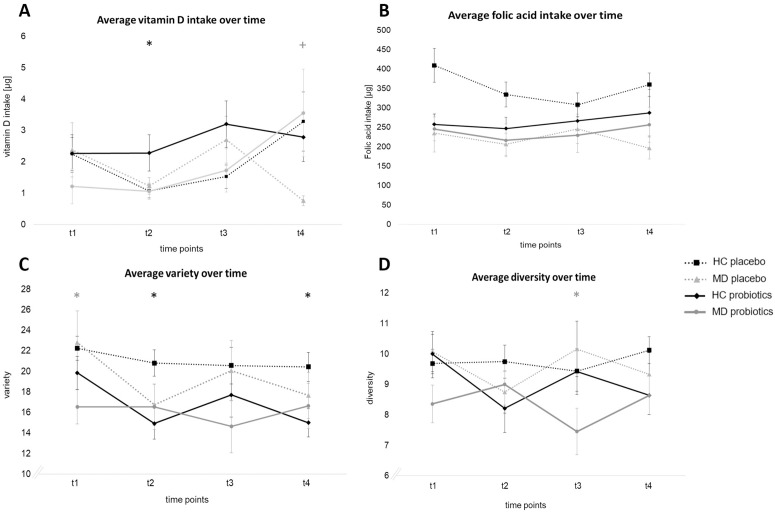
Average nutrition values for the four groups over time. **A) Vitamin D, B) Folic acid, C) Dietary variety, and D) Diversity.** *Note*. Error bars show the standard error. Asterisks show significant Bonferroni-corrected pairwise comparisons between *HC* (black) and *MC* (gray) groups; + represents a trend towards significance.

We found no significant main effect *time* (*p* = .063), *intervention* (*p* = .434), or *diagnosis* (*p* = .278), and no significant interactions between the factor pairs (all *p* > .157). However, we observed a significant interaction between *time x intervention x diagnosis* (*F*(2.33, 113.99) = 4.51, *p* = .009, *η*_*p*_*²* = .08). The results revealed a differentiated, time-dependent pattern of effects (see Fig 2A).

Among *HC*, we found a significant difference between intervention conditions at *t2*, with individuals in the *probiotic* group showing significantly higher values in vitamin D intake compared to those in the *placebo* group (*p* = .023).

In the *MD* group, a similar trend emerged at *t4*, with higher values under *probiotic* treatment than under *placebo*, although this difference did not reach significance (*p* = .058).

Comparisons between *MD* and *HC* within each intervention condition revealed that under *probiotic* treatment, *MD* had significantly lower values than *HC* at *t2* (*p* = .036). Under *placebo*, a similar group difference was observed at *t4*, but did not reach statistical significance (*p* = .062).

#### Folic acid.

Our data showed no significant effect of *time* (*p* = .199), or *intervention* (*p* = .220), but a significant effect for *diagnosis* (*F*(1, 48) = 6.56, *p* = .014, *η*_*p*_*²* = .12), suggesting that *HC* had a higher intake of folic acid than patients with *MD* over all four time points. There were no significant interactions between factor pairs (all *p* > .158) or between *time x intervention x diagnosis* (*p* = .219), see Fig 2B.

#### Fiber.

We observed a significant main effect of *time* (*F*(3, 147) = 3.38, *p* = .020, *η*_*p*_*²* = .06), but no significant differences for *intervention* (*p* = .237) and *diagnosis* (*p* = .203). Moreover, there were no significant effects for interactions between factor pairs (all *p* > .169) or the interaction *time x intervention x diagnosis* (*p* = .290). These findings indicate a significant decrease in fiber intake between *t1* and *t2*, independent of groups.

#### Variety.

We found a significant effect *time* (*F*(3, 147) = 6.44, *p* < .001, *η*_*p*_*²* = .12) and *intervention* (*F*(1, 49) = 5.22, *p* = .027, *η*_*p*_*²* = .10), but no effect *diagnosis* (*p* = .300) or between the factor pairs (all *p* > .611). Our data also showed a significant interaction *time x intervention x diagnosis* (*F*(3, 147) = 4.63, *p* = .004, *η*_*p*_*²* = .09), see Fig 2C.

In the *MD* group, participants receiving *placebo* reported significantly higher scores in their dietary variety at *t1* compared to those receiving the *probiotic* (*p* = .041).

In the *HC* group, significant differences between intervention conditions were found at *t2* and *t4*, with the *placebo* group again showing higher values than the *probiotic* group (*p* = .016 and *p* = .031, respectively).

These results suggest that across groups and most time points, the *probiotic* intervention was associated with lower outcome scores.

#### Diversity.

Our data showed no significant effect for *time* (*p* = .343), *intervention* (*p* = .097), or *diagnosis* (*p* = .439) and no significant interaction between factor pairs (all *p* > .632). We found a significant interaction between *time x interaction x diagnosis* (*F*(3, 147) = 5.16, *p* = .002, *η*_*p*_*²* = .10), see Fig 2D.

Post-hoc comparisons revealed that, within the *MD* group, participants receiving *placebo* had significantly higher diversity scores than those receiving *probiotics* at time point 3 (*p* = .030). No other time points in this group showed statistically significant differences.

These results indicate that probiotic supplementation may have led to lower scores specifically in the *MD* group at time point 3, with no such effects observed in the *HC* group.

#### Drop-out analysis.

Chi-square tests of independence showed no significant association between diagnostic group and study inclusion (χ²(1) =.54, p = .463, φ = .079), between smoking status and study inclusion (χ²(1) = 1.49, p = .222, φ = .132), and sex and study inclusion (χ²(1) = 1.59, p = .207, φ = .136). All expected cell frequencies met the assumptions for the test. Independent-samples t-tests between included and excluded participants showed no significant differences for height, t(84) =.20, p = .840; depressive symptoms measured by the HAMD, t(82) =.02, p = .987, or depressive symptoms measured by the BDI, t(79) = −.81, p = .420. Mann–Whitney-U tests further indicated no significant differences between included and excluded participants in age (U = 798.00, Z = −.68, p = .500), BMI (U = 748.00, Z = −1.12, p = .264), or weight (U = 700.50, Z = −1.34, p = .183).

The drop-out analysis indicated that included and excluded participants did not differ significantly in any of the examined variables.

## Discussion

### Summary of findings

The present study aimed to determine whether daily supplementation with probiotics over a three-month period influences dietary intake patterns in participants with *MD* and *HC*. Sixteen predefined nutritional parameters were analyzed to assess potential group differences and temporal patterns. The probiotic intervention did not result in consistent changes in macro- or micronutrient intake. Instead, exploratory time- and group-dependent patterns emerged for selected measures, including vitamin D, folic acid, fiber, and dietary variety and diversity. Overall, the findings indicate that potential probiotic-associated effects on dietary intake in this sample were relatively small, heterogeneous, and context dependent and should be interpreted cautiously as hypothesis- generating rather than confirmatory.

### Vitamin D

Vitamin D intake showed an exploratory three-way interaction (*time × diagnosis × intervention*). Among HC, probiotic supplementation was associated with significantly higher vitamin D intake at one week after intervention start (t2) compared to placebo; however, this difference was primarily driven by a marked decrease in the HC placebo group, while intake in the HC probiotic group remained relatively stable across time. In contrast, participants with major depression (MD) showed a delayed pattern: vitamin D intake in the probiotic group increased steadily throughout the study, culminating in higher intake after the three-month intervention (t4) compared to the MD placebo group. Notably, this difference reached only trend-level significance (p = .058) and was not consistent across groups and time points. Taken together, these findings suggest a possible diagnosis- and time-dependent pattern, but should be interpreted with caution given their conditional nature and limited robustness.

One possible, albeit speculative, explanation is that probiotic supplementation may indirectly influence dietary behavior via gut–brain axis pathways affecting mood, motivation, and appetite regulation [86–88]. Such mechanisms could be particularly relevant in depression, where reduced motivation and suboptimal dietary patterns may limit nutrient intake, although direct evidence for this pathway is currently lacking since the present study did not assess appetite, food motivation, gastrointestinal symptoms, vitamin D status, or microbiome composition directly.

Given the small subgroup sizes, these patterns should be interpreted with caution and should be viewed as descriptive trends intended to inform future studies rather than evidence of a reliable intervention effect. In particular, future well-powered trials should combine microbiome profiling, validated measures of appetite and food preferences, and biomarker-based vitamin D assessments over extended follow-up periods to confirm whether probiotic supplementation can support sustained improvements in vitamin D intake and status, particularly in individuals with major depression.

### Folic acid

Folic acid intake was consistently lower in *MD* compared to *HC* across all time points, independent of intervention. Notably, the two groups (*HC, MD*) already showed significant differences at baseline. This observation is consistent with prior findings linking depression to suboptimal nutritional status, including deficiencies in B vitamins such as folate [89–91]. Folate plays a critical role in one-carbon metabolism and methylation pathways, acting as an essential cofactor for the conversion of homocysteine to methionine and for the synthesis of S-adenosylmethionine (SAM), the universal methyl donor involved in DNA, RNA, neurotransmitter, and phospholipid methylation. Impaired methylation capacity has been implicated in the pathophysiology of depression, as it can disrupt monoamine neurotransmitter synthesis and gene expression relevant to mood regulation. In this context, the use of bioactive L-methylfolate as an adjunctive treatment in depression has been recommended in the World Federation of Societies of Biological Psychiatry (WFSBP) guidelines on nutraceuticals and phytoceuticals for depression [60]; particularly in individuals with low folate status or genetic variants affecting folate metabolism (e.g., MTHFR polymorphisms) [91,92].

### Fiber

Fiber intake declined significantly after one week (*t2*), regardless of *intervention* or *diagnosis*. These findings may reflect short-term reactivity to study procedures (e.g., dietary recording or stress from HRV monitoring), rather than intervention effects.

### Dietary variety

Dietary variety also showed an exploratory three-way interaction (*time × diagnosis × intervention*). In healthy controls, higher variety scores were observed in the *placebo* group at selected time points (*t2, t4*) compared to the *probiotic* group, suggesting a potential reduction in dietary variety due to probiotic supplementation. In the depressed group, participants receiving the placebo reported significantly higher variety scores at time point *t1* compared to those in the probiotic group. However, since baseline comparisons already showed significant differences between intervention groups prior to the start of the intervention, with the *placebo* group reporting higher variety than the *probiotic* group, group effects may not be attributable to the intervention itself.

Regarding dietary variety and diversity, both indicators of overall diet quality, our results consistently showed lower scores in the *probiotic* group at certain time points. This pattern cannot be explained by a clear underlying mechanism based on the present data. One possible interpretation is that these findings reflect subtle behavioral changes rather than direct effects on nutrient intake per se. For example, participants may have relied on a narrowing of dietary choices during probiotic supplementation, potentially influenced by factors such as perceived gastrointestinal effects, routine stabilization, or changes in food-related decision-making. However, these interpretations remain speculative and cannot be directly tested within the current study design.

A reduction in dietary variety and diversity has previously been associated with a shift in gut microbiota composition induced by the intake of live microorganisms [93]. Although microbiome data were not included in the present analysis, findings from the parent trial [69] provide relevant contextual information. In that study, probiotic supplementation was associated with functional changes, such as altered vagal nerve activity, despite the absence of significant changes in overall microbial diversity. This dissociation between functional effects and compositional stability suggests that probiotic effects may not necessarily be reflected in global diversity measures, but may instead operate through more subtle functional or signaling pathways. In this context, the observed changes in dietary variety and diversity may reflect indirect behavioral modulation rather than direct microbiome restructuring. It is conceivable that the altered microbiome required a less diverse external nutrient input, which may have contributed to a decline in these measures.

Given the exploratory nature of these findings, the small sample size, and the presence of baseline imbalances, the observed patterns should be interpreted with caution.

Future studies integrating microbiome profiling, appetite and food preference assessments and objective diet quality measures should replicate and extend these findings by clarifying the mechanisms underlying this pattern.

### Adherence

Participants receiving probiotics reported significantly higher subjective adherence than those receiving placebo, despite the blinded study design. This could raise concerns about potential unblinding. However, several factors argue against systematic unblinding. Both probiotic and placebo sachets were identical in appearance, consistency, taste, and mode of administration, and no participant reported awareness of group allocation. The observed difference may therefore reflect subtle differences in perceived tolerability, gastrointestinal comfort, or general well-being that may have influenced motivation for consistent intake without necessarily revealing group assignment. Alternatively, nonspecific factors such as random variation in motivation, perceived well-being, or daily routines may have contributed to the difference. The absence of adherence differences between MD and HC participants further suggests that diagnosis did not systematically influence compliance.

### Limitations

Several limitations and potential sources of bias should be considered when interpreting the findings of this pilot study. The primary limitation is the assessment of dietary intake for which we used a self-report food record, which can be inherently susceptible to recall errors and social desirability bias. Specifically, participants may have underreported or misreported their consumption due to recall difficulties or discomfort about disclosing certain eating patterns. Moreover, although the Vienna Food Record is a validated prospective instrument typically applied over multiple days to approximate habitual intake, dietary assessment in the present study was restricted to a single 24-hour record at each time point. This decision was driven by feasibility considerations in a clinically vulnerable population with major depressive disorder, where multi-day dietary recording is often associated with reduced adherence due to symptom-related burden (e.g., impaired concentration and motivation). To reduce drop-out, dietary assessment was therefore restricted to one day in close temporal proximity to biological sampling. While this represents a pragmatic compromise, it precludes conclusions about habitual intake and should be considered a methodological learning point for future studies.

Although microbiome data were not included in the present analysis, results from the parent trial [69] provide important contextual information. Notably, probiotic supplementation was associated with functional changes, such as altered vagal nerve activity, despite the absence of significant changes in overall microbial diversity. This dissociation between functional effects and compositional stability suggests that probiotic effects may not necessarily be reflected in global diversity measures. However, as microbiome data were not linked to dietary outcomes in the present analysis, these interpretations remain speculative and require confirmation in future studies integrating microbiome, behavioral, and nutritional dataIn addition, no objective mechanistic measures (e.g., appetite-regulating hormones, neural or behavioral endpoints) were included. Consequently, the study does not allow conclusions regarding the biological pathways underlying the observed changes in dietary behavior. A key limitation is that the relatively small sample size (N = 53) substantially limited statistical power and generalizability. This constraint is especially critical for subgroup and interaction analyses, which are highly susceptible to spurious findings in small samples and should therefore be regarded as exploratory. The limited power may also have precluded detection of small to moderate effects. For example, the observed increase in vitamin D intake in the probiotic group among participants with major depression at *t4* did not reach statistical significance (*p* = .058), despite mirroring patterns seen in healthy controls at earlier time points.

In addition, only four time points were used to assess dietary intake, including baseline and three follow-ups during the intervention period. While this design balances data resolution with participant burden, it may have missed finer fluctuations in dietary patterns and, more importantly, long-term effects, especially with nutrients like vitamin D. Thus, the intervention period of three months provides only a limited view of longer-term changes in dietary patterns and gut microbiota composition. Extending the observation period or including post-intervention follow-up assessments could offer a clearer picture of the stability and persistence of any observed changes.

Baseline differences between groups in certain dietary variables (i.e., folic acid intake and dietary variety) represent an additional limitation. Such imbalances may influence the interpretation of observed changes, as differences at later time points could partly reflect pre-existing group differences rather than intervention effects. Although the applied statistical models account for within-subject changes over time, residual confounding due to baseline variability cannot be ruled out. Another factor to consider is the Hawthorne effect, as participants’ awareness of being part of a study may have influenced their dietary choices, consciously or unconsciously [94]. While this effect is difficult to quantify, it may have led to temporary behavior changes that do not reflect long-term habits.

Finally, dietary supplement use of participants was not assessed. This could have introduced unmeasured variability in nutrient intake and confounded intervention effects.

Taken together, these limitations underscore that the present findings should be interpreted as descriptive and hypothesis-generating rather than in an inferential manner. They also highlight important methodological considerations for future, adequately powered trials, including improved control of baseline differences, repeated dietary assessments of habitual intake, and the inclusion of objective biomarkers to test these hypotheses.

## Conclusions

Although probiotics have been hypothesized to influence dietary behavior through gut–brain axis pathways, the absence of assessments of appetite-regulating hormones, neurotransmitters, neuroimaging, and microbiome composition in the present study limits mechanistic interpretation of the observed changes in dietary variety and diversity. While findings from the parent trial [69] suggest that probiotic supplementation induce functional changes (e.g., altered vagal nerve activity) without detectable alterations in overall microbial diversity, the present results should be regarded as exploratory and hypothesis-generating rather than as evidence of a confirmed biological effect.

In summary, while probiotic supplementation did not lead to consistent changes in nutrient intake across all participants, exploratory, group-specific, and time-dependent patterns were observed for selected parameters – including vitamin D intake as well as dietary variety and diversity. Given the pilot nature of the study, baseline differences between groups, reliance on self-reported dietary data, and limited statistical power, these patterns should be interpreted as descriptive and indicative rather than as evidence of causal intervention effects. Future research should replicate and extend these preliminary results in larger, adequately powered samples. Such studies should integrate objective biomarkers of nutrient status, systematic assessment of supplement use, and comprehensive microbiome profiling to elucidate whether and how specific probiotic formulations may meaningfully influence dietary behavior or nutrient status in clinical and non-clinical populations. Taken together, the present findings provide preliminary methodological and descriptive insights to guide the design of future investigations into the complex interplay between probiotics, diet, and mental health.

## Supporting information

S1 File 7_Abschlussbericht. (PDF)

S2 File 7_Final report. (PDF)

S3 File CONSORT_2025_editable_checklist_LGS. (DOCX)

S4 File EK_Protokoll_V1.2_03072021 en-GB 1. (PDF)

S5 File EK_Protokoll_V1.2_03072021. (PDF)

S6 File EK-Vote_till_2022-06-30. (PDF)

S7 File EK-Vote_till_2023_06_30. (PDF)

S8 File EK-Vote_till_2024-06-30. (PDF)

S9 File EK-Votum_bis_2022-06-30. (PDF)

S10 File EK-Votum_bis_2023-06-30. (PDF)

S11 File EK-Votum_bis_2024-06-30. (PDF)
