# Supplementary material for: Do probiotics modulate dietary intake? Pilot data from a randomized controlled sub-study of the ProBioHRV clinical trial in patients with depression and healthy controls
Source: PLoS One. 2026 Jun 23;21(6):e0350801. doi: 10.1371/journal.pone.0350801 (PMC13289889; doi:10.1371/journal.pone.0350801)
Supplement: S1 File — (PDF) [file pone.0350801.s001.pdf]

Bericht

Formular für Zwischen- und Abschlussberichte.

Alle Angaben sind für den **Zeitraum seit Studienbeginn** zu machen!

Gültig für: ☐ **Prüfer/in** (Pkt. 2.2) ☐ **gesamte Studie** *(bitte Zutreffendes ankreuzen)*

Version 2.1 vom 31.01.2022 **Bitte immer die *aktuelle* Version verwenden (http://ethikkommissionen.at)!**

Adresse der Ethikkommission (optional)	Raum für Eingangsstempel, EK-Nummer, etc. Bitte Freilassen!
EK der MedUni Graz	

☐ **1.1 JÄHRLICHER ZWISCHENBERICHT / ANTRAG AUF VERLÄNGERUNG**

☒ **1.2 MELDUNG DER BEENDIGUNG / ABSCHLUSSBERICHT**

2. Angaben zur Studie:

2.1 Antragsteller/in: **Res. Prof. PD DDr. Sabrina Mörkl**

2.2 Prüfer/in: **Res. Prof. PD DDr. Sabrina Mörkl**

2.3 Studienkurztitel: **ProBioHRV Studie**

2.4 EK-Nummer: **33-227 ex 20/21** 2.5 Datum des Votums: **08.02.2021**

3. Angaben zum Studienverlauf:

3.1 Wurde die Studie begonnen? ☒ ja ☐ nein

Wenn **nein**, Begründung:

3.2 Zahl der rekrutierten Patient*innen / Proband*innen: **86**

3.3 Zahl der Patient*innen / Proband*innen, die die Studie beendet haben: **77**

3.4 Zahl der Studienabbrüche: **9**

3.5 Zahl der ☐ SAEs, SADE ☐ SUSARs *(bitte Zutreffendes ankreuzen)*: **0**

3.6 Status der Studie:
- ☐ (3.6.1) läuft noch bis voraussichtlich (Datum)
- ☒ (3.6.2) planmäßig abgeschlossen am **10.10.2023** (Datum)
- ☐ (3.6.3) abgebrochen am (Datum)

(wenn abgebrochen, bitte unter Punkt 4 kommentieren!)

4. Ergebnisse und Schlussfolgerungen (ggf. auf Beiblatt):

siehe Beiblätter

☐ Ich beantrage die Verlängerung der Gültigkeit des Votums.

5. Unterschrift

[Unterschrift] 24.06.24

Unterschrift (Prüfer/in bzw. Antragsteller/in) Datum

Abschlussbericht-Ethikkommission ProBioHRV

Abstract

Die Major Depression (MD) ist eine weit verbreitete Störung mit tiefgreifenden Auswirkungen auf das individuelle Wohlbefinden und die Gesellschaft. Der Vagusnerv ist eine entscheidende Komponente der Darm-Hirn-Achse, die die bidirektionale Kommunikation zwischen Darm und Gehirn ermöglicht. Aktuelle Meta-Analysen legen nahe, dass Probiotika antidepressive Wirkungen haben könnten, obwohl ihre genauen Mechanismen noch unklar sind. Tierversuche haben gezeigt, dass die Supplementierung mit Probiotika die Vagalaktivierung verbessern kann, wobei Vagotomie diese Effekte blockiert. Diese Studie zielt darauf ab, die Auswirkungen eines Multi-Stamm-Probiotikums auf die Funktion des Vagusnervs bei Patienten mit MD und gesunden Kontrollen zu analysieren und postuliert eine signifikante Verbesserung durch die probiotische Intervention. An der Studie nahmen 43 Patienten mit MD und 43 gesunde Kontrollpersonen teil, die zweimal täglich ein Multi-Stamm-Probiotikum oder ein Placebo einnahmen. Serum- und Stuhlproben wurden zu Beginn, nach 7 Tagen, 28 Tagen und nach 3 Monaten gesammelt. Die vagale Nervenfunktion wurde mittels Herzfrequenzvariabilität (HRV) anhand eines 24-Stunden-EKGs beurteilt, zusammen mit entzündlichen Parametern und einer 16S-Analyse der Stuhlproben. Die Patienten, die Probiotika einnahmen, zeigten nach 3 Monaten signifikant verbesserte morgendliche vagale Funktion. Während sich die Zusammensetzung des Darmmikrobioms zu Beginn unterschied, hatte die Intervention insgesamt keinen Einfluss auf die Diversität. Allerdings zeigten depressive Teilnehmer nach 3 Monaten der probiotischen Intervention eine Zunahme der Christensellales und eine Abnahme von Ruminococcus. Diese Studie unterstreicht das potenzielle physiologische Wirken von Probiotika bei MD, möglicherweise durch die Stimulation des Vagusnervs.

Results

148 Personen wurden auf Eignung geprüft, wobei 62 Personen ausgeschlossen wurden, weil sie die Einschlusskriterien nicht erfüllten (n=55) oder zu weit vom Studienort entfernt lebten. 86 Personen (40 Patienten mit Depressionen und 46 gesunde Kontrollpersonen) gaben ihre schriftliche Einwilligung und wurden randomisiert einer Interventions- und Placebogruppe zugeteilt (siehe Abbildung 2) und absolvierten mindestens den Baseline-Besuch (t0, n=86; t1, n=84; t2, n=78; t3, n=77). Tabelle 1 gibt einen Überblick über die Studienpopulation. Figure 1 gibt das CONSORT Flow Diagram wieder.

Es gab keine signifikanten Unterschiede in klinischen Variablen wie Alter, Geschlecht, Gewicht (Baseline), und Größe (Baseline) zwischen den Teilnehmern mit Depressionen und den gesunden Kontrollen.

Jedoch gab es signifikant mehr Raucher in der Depressionsgruppe im Vergleich zur Kontrollgruppe (X2 (1, n = 86) = 5,779, p = 0,016). Weiterhin gab es keine signifikanten Unterschiede bezüglich Alter, Geschlecht, Gewicht, Größe und Raucherstatus zwischen der Probiotika- und Placebogruppe.

In der Probiotika-Gruppe gab es keine Unterschiede in klinischen Variablen (Gewicht, Größe, BMI, Blutdruck, Puls) zwischen Patienten mit Depressionen und gesunden Kontrollen, außer dass es signifikant mehr Frauen in der Untergruppe der Depressionen gab (X2 (1, n = 43) = 4,740, p = 0,029). Außerdem gab es signifikant mehr Raucher in der Untergruppe der Depressionen (X2 (1, n = 43) = 5,874, p = 0,015).

Wie erwartet, gab es signifikante Unterschiede in den Baseline-Werten zwischen Patienten mit Depressionen und gesunden Kontrollen bezüglich Depressions- und Stressscores (p < 0,001), aber keine signifikanten Unterschiede in den Depressions- und Stresslevels zwischen den Baseline-Werten der Probiotika- und Placebogruppe für depressive Patienten und gesunde Kontrollen separat.

	**Probiotic Group**			**Placebo Group**		
	**Depression (n = 20)**	**Healthy Control (n = 23)**	**p-value**	**Depression (n = 20)**	**Healthy Control (n = 23)**	**p -value**
**Sex (female)**	16	11	0.029	15	17	0.935
**smoker**	6	4	0.329	8	2	0.015
	mean (SD)	mean (SD)		mean (SD)	mean (SD)	
**Age (years)**	32.65 (8.83)	35.30 (10.10)	0.407	37.5 (14.73)	37.13 (14.68)	0.884
**BDI (t0)**	17.16 (11.68)	3.95 (4.39)	<0.001	21.89 (10.62)	3.87 (3.44)	<0.001
**HAMD (t0)**	18.10 (10.91)	2.05 (1.40)	<0.001	18.42 (9.44)	1.70 (1.55)	<0.001
**PSS Score (t0)**	31.45 (7.31)	21.62 (5.53)	<0.001	34.94 (6.14)	20.45 (5.37)	<0.001
**Weight [kg]**	75.00 (18.12)	68.32 (14.22)	0.183	72.35 (20.51)	67.98 (10.97)	0.383
**Height [m]**	1.69 (0.84)	1.71 (0.92)	0.578	1.69 (0.96)	1.70 (0.90)	0.828
**BMI [kg/m²]**	26.00 (5.95)	23.14 (3.26)	0.108	24.99 (6.66)	23.36 (3.33)	0.441

Table 1. Baseline Characteristics. Notes. BDI = Beck Depression Inventory; HAMD = Hamilton Depression Scale, RR= blood pressure, BMI= Body mass index.

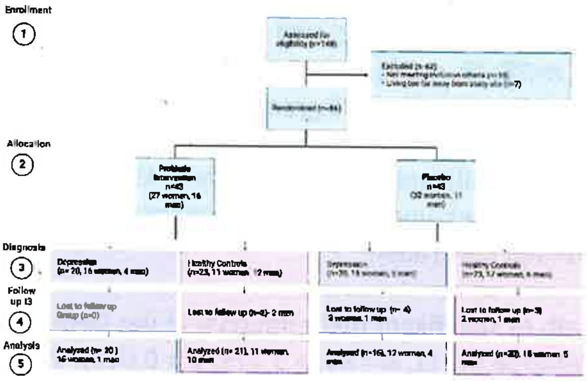

Figure 1. Consort Flow Diagram

<u>HRV</u>

24-Stunden-HRV-Messungen

Die wiederholte Messungen ANOVA für die 24-Stunden-Mittelherzfrequenz (meanHR) ergab eine signifikante Interaktion zwischen Zeit und Probiotika, $F(3, 198) = 3{,}244$, $p = .027$, $\eta^2 = 0{,}047$, sowie zwischen Zeit und Diagnose, $F(3, 195) = 4{,}351$, $p = .021$, $\eta^2 = 0{,}050$. Depressive Patienten zeigten im Vergleich zu gesunden Kontrollen signifikant höhere Herzfrequenzen ($F(1, 66) = 18{,}048$, $p < .001$) (siehe Abbildung 3).

Für pNN50, RMSSD und SDNN über 24 Stunden zeigte sich ein signifikanter Effekt der Diagnose ($F(1, 65) = 6{,}871$, $p = 0{,}011$; $F(1, 66) = 9{,}937$, $p = 0{,}002$; und $F(1, 66) = 14{,}257$, $p < 0{,}001$, jeweils), wobei Patienten mit Depressionen signifikant niedrigere Werte als gesunde Kontrollen aufwiesen. Es gab jedoch keine signifikanten Unterschiede zwischen der Probiotika- und Placebogruppe und über die Zeitpunkte hinweg. Weiterhin gab es keine signifikanten Unterschiede in Bezug auf lnHF. In Bezug auf 24-Stunden logRSA zeigte die wiederholte Messungen ANOVA eine signifikante Interaktion zwischen Zeit und Diagnose ($F (3, 198) = 2{,}791$, $p = 0{,}042$, $\eta^2 = 0{,}041$).

Morgendliche und nachmittägliche HRV-Messungen

In der wiederholten Messungen ANOVA zeigte die Herzfrequenz am Morgen eine signifikante Interaktion von Zeit und Probiotikaeinnahme ($F(3, 192) = 3{,}987$, $p = .009$, $\eta^2 = 0{,}059$) sowie eine Interaktion von Zeit*Probiotika*Diagnose ($F(3, 192) = 6{,}062$, $p < .001$, $\eta^2 = 0{,}087$). Depressive Patienten, die ein Probiotikum einnahmen, hatten nach 3 Monaten signifikant niedrigere Herzfrequenzen im Vergleich zu denen, die ein Placebo einnahmen. Auch zwischen Teilnehmern mit Depressionen und gesunden Kontrollen gab es einen signifikanten Unterschied (($F(1, 64) = 9{,}722$, $p = 0{,}003$)), wobei Patienten signifikant höhere Herzfrequenzen im Vergleich zu gesunden Kontrollen zeigten. Für die nachmittäglichen Werte zeigte die Herzfrequenz keine signifikanten Interaktionen für die Zeit, aber der signifikante zwischen-subjekt-Effekt der Diagnose blieb bestehen (($F(1, 64) = 11{,}723$, $p = 0{,}001$)).

RMSSD am Morgen zeigte eine signifikante Interaktionseffekt von Zeit*Probiotika*Diagnose, $F(3, 192) = 4{,}181$, $p = .007$, $\eta^2 = 0{,}061$; sowie einen signifikanten Haupteffekt der Diagnose, wobei depressive Patienten im Vergleich zu gesunden Kontrollen niedrigere RMSSD-Werte aufwiesen ($F(1, 64) = 9{,}919$, $p = 0{,}004$). Für den Nachmittag blieb nur der signifikante Unterschied zwischen den Diagnosen erhalten (($F(1, 66) = 7{,}795$, $p = 0{,}007$))

In der wiederholten Messungen ANOVA zeigte lnHF am Morgen eine signifikante Interaktion von Zeit*Probiotika*Diagnose ($F(2{,}726, 192) = 2{,}986$, $p = 0{,}037$, $\eta^2 = 0{,}016$). Ebenso gab es einen signifikanten Unterschied zwischen den Teilnehmern bezüglich der Diagnose (($F(1, 64) = 6{,}800$, $p = 0{,}011$)). Für den Nachmittag blieb nur der signifikante Unterschied zwischen den Diagnosen erhalten (($F(1, 66) = 6{,}394$, $p = 0{,}014$)

Für logRSA am Morgen zeigte sich ein signifikanter Interaktionseffekt von Zeit*Probiotika und Diagnose, $F(3, 192) = 3{,}744$, $p = 0{,}012$, $\eta^2 = 0{,}055$). Ebenso gab es einen signifikanten Unterschied in logRSA zwischen Patienten mit Depressionen und gesunden Kontrollen ($F(1, 64) = 8{,}799$, $p = 0{,}004$). Für den Nachmittag blieb nur der signifikante Unterschied zwischen den Diagnosen erhalten ($F(1, 66) = 8{,}860$, $p = 0{,}004$).

In Bezug auf die Herzfrequenz gab es nur einen signifikanten Haupteffekt für die Diagnose, wobei Teilnehmer mit Depressionen während ruhigen Schlafes (F(1, 68) = 16,479, p = 0,001) und unruhigen Schlafes (F(1, 68) = 13,402, p = 0,001) eine höhere Herzfrequenz hatten.

Für pNN50 wurde nur ein signifikanter Unterschied zwischen Patienten mit Depressionen und gesunden Kontrollen während des ruhigen Schlafes gefunden (F(1, 68) = 8,301, p = 0,006) und während des unruhigen Schlafes (F(1, 68) = 8,204, p = 0,006).

Für RMSSD im ruhigen Schlaf gab es eine signifikante Interaktion von Zeit*Diagnose (F(3, 204) = 3,075, p = 0,029, η^2 = 0,043) und einen signifikanten Unterschied zwischen Patienten mit Depressionen und gesunden Kontrollen (F(1, 68) = 6,753, p = 0,011), während es im unruhigen Schlaf nur einen signifikanten Unterschied zwischen Patienten und Kontrollen gab (F(1, 68) = 9,155, p = 0,003).

Auch bei den HF-Messungen im ruhigen und unruhigen Schlaf gab es einen signifikanten Unterschied zwischen Patienten und Kontrollen (ruhiger Schlaf: F(1, 68) = 6,919, p = 0,011; unruhiger Schlaf: F(1, 68) = 7,787, p = 0,007).

Hinsichtlich logRSA wurden ebenfalls signifikante Unterschiede zwischen Patienten und Kontrollen im unruhigen Schlaf (F(1, 68) = 8,204, p = 0,006) und im ruhigen Schlaf (F(1, 68) = 8,031, p = 0,006) festgestellt.

Für SDNN im ruhigen Schlaf gab es eine signifikante Interaktion von Zeit*Diagnose (F(3, 204) = 2,777, p = 0,049, η^2 = 0,039) und einen signifikanten Unterschied zwischen Patienten und Kontrollen (F(1, 68) = 7,172, p = 0,009). Im unruhigen Schlaf gab es nur einen signifikanten Unterschied hinsichtlich der Diagnose (F(1, 68) = 12,336, p < 0,001).

Mikrobiom

nsgesamt standen 313 Stuhlproben zur Verfügung (143 Proben von Patienten mit Depressionen und 170 Proben von gesunden Kontrollen, 157 Proben von Teilnehmern, die Probiotika erhielten, und 156 Proben von Teilnehmern, die ein Placebo erhielten), wobei Proben von 66 von 86 Teilnehmern (76,74%) für alle Zeitpunkte verfügbar waren.

In Bezug auf die Chao-1 Diversität (F(1, 62) = 6,094, p = 0,017), Anzahl der beobachteten Arten (F(1, 62) = 7,865, p = 0,007), Simpson-Index (F(1, 62) = 5,119, p = 0,027) und Shannon-Index (F(1, 62) = 9,555, p = 0,003) zeigte sich ein signifikanter zwischen-subjekt-Effekt für die Diagnose, wobei depressive Patienten eine niedrigere Alpha-Diversität aufwiesen. Die Alpha-Diversität wurde jedoch durch die Einnahme von Probiotika oder im Verlauf der Zeit nicht signifikant verändert.
Es gab einen signifikanten Unterschied der Beta-Diversität zu Baseline-Zeitpunkten zwischen Patienten mit Depressionen und gesunden Kontrollen (p = 0,001). PCoA und Redundanzanalyse zeigten keinen signifikanten Effekt der Probiotika auf die Gesamtkomposition des Mikrobioms im Verlauf der Zeit.

Weitere Parameter betreffend Fragebögen und Laborparameter befinden sich derzeit noch in Auswertung.

Schlussfolgerungen:

Die vorliegende Studie liefert wertvolle Einblicke in die potenziellen Wirkmechanismen von Probiotika bei der Major Depression (MD). Die Ergebnisse deuten darauf hin, dass die tägliche Einnahme eines Multi-Stamm-Probiotikums über einen Zeitraum von drei Monaten die morgendliche vagale Funktion bei Patienten mit MD signifikant verbessern kann. Diese Verbesserung könnte auf eine gesteigerte Vagalaktivierung zurückzuführen sein, wie sie in Tierstudien beobachtet wurde, und unterstützt die Hypothese, dass Probiotika möglicherweise durch die Stimulation des Vagusnervs antidepressive Effekte vermitteln.

Obwohl sich die Zusammensetzung des Darmmikrobioms zwischen depressiven Patienten und gesunden Kontrollen zu Beginn der Studie unterschied, zeigte die probiotische Intervention keinen signifikanten Einfluss auf die Gesamtdiversität des Mikrobioms. Interessanterweise wurden jedoch spezifische Veränderungen im Mikrobiom festgestellt, insbesondere eine Zunahme von Christensellales und eine Abnahme von Ruminococcus bei den depressiven Teilnehmern nach drei Monaten der probiotischen Behandlung.

Diese Ergebnisse legen nahe, dass Probiotika potenziell therapeutische Optionen für die Behandlung von MD darstellen könnten, indem sie gezielt auf die physiologischen Wechselwirkungen zwischen Darm und Gehirn wirken. Zukünftige Forschung sollte die genauen Mechanismen weiter untersuchen und größere, randomisierte kontrollierte Studien durchführen, um die Langzeiteffekte und die klinische Relevanz dieser Interventionen besser zu verstehen.
