# Supplementary material for: Do probiotics modulate dietary intake? Pilot data from a randomized controlled sub-study of the ProBioHRV clinical trial in patients with depression and healthy controls
Source: PLoS One. 2026 Jun 23;21(6):e0350801. doi: 10.1371/journal.pone.0350801 (PMC13289889; doi:10.1371/journal.pone.0350801)
Supplement: S2 File — (PDF) [file pone.0350801.s002.pdf]

Report

Form for interim and final reports.

All information must be provided for the period since the start of the

Valid for: Examiner (point 2.2) Entire study *(please tick the applicable box)*

Version **2.1 as of 31 January 2022** Please always use the *latest* version (http://ethikkommissionen.at)!

Address of the ethics committee (optional)	Space for receipt stamp, EC number, etc. Please leave blank!
Ethics Committee of<br>MedUni Graz	

1.1 ANNUAL INTERIM REPORT / APPLICATION FOR EXTENSION

@ **1.2 NOTIFICATION OF TERMINATION / FINAL REPORT**

2. Study details:

2.1 Applicant: **Res. Prof.** PD **DDr. Sabrina Märkl**

2.2 Examiner: Res. Prof. PD DDr. Sabrina Mörkl

2.3 Study title: **ProBioHRV study**

2.4 EK number: 33-227 ex 20/21 2.5 Date of vote: 08.02.2021

3. Information on the course of the study:

3.1 Has the study been started? @ yes No

If no, reason:

3.2 Number of patients/subjects recruited: 86

3.3 Number of patients/subjects who completed the study: 77

3.4 Number of study dropouts: 9

3.5 Number of SAEs, SADEs SUSARs *(please tick as applicable):* 0

3.6 Status of the study: (3.6.1) still ongoing, expected to continue until (date)

§§ (3.6.2) completed as planned on **10 October 2023** (date)

(3.6.3) discontinued on (date)

(if discontinued, please comment under point 4!)

4. Results and conclusions (on supplementary sheet if necessary):

see supplementary sheets

☐ I request an extension of the validity of the vote.

5. Signature

Final report – Ethics Committee ProBioHRV

Abstract

Major depression (MD) is a widespread disorder with profound effects on individual well-being and society. The vagus nerve is a crucial component of the gut-brain axis, enabling bidirectional communication between the gut and the brain. Recent meta-analyses suggest that probiotics may have antidepressant effects, although their exact mechanisms remain unclear. Animal studies have shown that probiotic supplementation can improve vagal activation, with vagotomy blocking these effects. This study aims to analyse the effects of a multi-strain probiotic on vagus nerve function in patients with MD and healthy controls and postulates a significant improvement through probiotic intervention. The study included 43 patients with MD and 43 healthy controls who took a multi-strain probiotic or a placebo twice daily. Serum and stool samples were collected at baseline, after 7 days, 28 days, and after 3 months. Vagal nerve function was assessed using heart rate variability (HRV) based on a 24-hour ECG, along with inflammatory parameters and a 16S analysis of stool samples. Patients taking probiotics showed significantly improved morning vagal function after 3 months. While the composition of the gut microbiome differed at baseline, the intervention had no overall effect on diversity. However, depressed participants showed an increase in Christensellales and a decrease in Ruminococcus after 3 months of probiotic intervention. This study highlights the potential physiological effects of probiotics in MD, possibly through stimulation of the vagus nerve.

Results

148 individuals were screened for eligibility, with 62 individuals excluded because they did not meet the inclusion criteria (n=55) or lived too far from the study site. Eighty-six individuals (40 patients with depression and 46 healthy controls) provided written consent and were randomly assigned to an intervention or placebo group (see Figure 2) and completed at least the baseline visit (t0, n=86; t1, n=84; t2, n=78; t3, n=77). Table 1 provides an overview of the study population. Figure 1 shows the CONSORT flow diagram.

There were no significant differences in clinical variables such as age, gender, weight (baseline), and height (baseline) between participants with depression and healthy controls.

However, there were significantly more smokers in the depression group compared to the control group (X2 (1, n = 86) = 5.779, p = 0.016). Furthermore, there were no significant differences in age, gender, weight, height, and smoking status between the probiotic and placebo groups.

In the probiotic group, there were no differences in clinical variables (weight, height, BMI, blood pressure, pulse) between patients with depression and healthy controls, except that there were significantly more women in the depression subgroup (X2 (1, n = 43) = 4.740, p = 0.029). In addition, there were significantly more smokers in the depression subgroup (X2 (1, n = 43) = 5.874, p = 0.015).

As expected, there were significant differences in baseline scores between patients with depression and healthy controls in terms of depression and stress scores (p < 0.001), but no significant differences in depression and stress levels between the baseline scores of the probiotic and placebo groups for depressed patients and healthy controls separately.

	Probiotic Group			Placebo Group		
	**Depression** (n = 20)	Healthy **Control** (n = 23)	**P** value	**Depression** (» = zo)	Healthy **Control** (» = :zs)	P **-value**
Sex (female)	16	11	0.029	15	17	0.935
smoker	6	4	0.329	8	2	0.015
	mean (SD)	mean (SD)		mean (SD)	mean (SD)	
Age (years)	32.65 (8.83)	35.30 (10.10)	0.407	37.5 (14.73)	37.13 (14.68)	0.884
BDI (t0)	17.16 (11.68)	3.95 (4.39)	<0.001	21.89 (10.62)	3.87 (3.44)	<0.001
HAMD (t0)	18.10 (10.91)	2.05 (1.40)	<0.001	18.42 (9.44)	1.70 (1.55)	<0.001
PSS score (t0)	31.45 (7.31)	21.62 (5.53)	<0.001	34.94 (6.14)	20.45 (5.37)	<0.001
Weight [kg]	75.00 (18.12)	68.32 (14.22)	0.183	72.35 (20.51)	67.98 (10.97)	0.383
Height [m]	1.69 (0.84)	1.71 (0.92)	0.578	1.69 (0.96)	1.70 (0.90)	0.828
BMI [kg/m^2]	26.00 (5.95)	23.14 (3.26)	0.108	24.99 (6.66)	23.36 (3.33)	0.44

Table 1. Baseline Characteristics. Notes. BDI = Beck Depression Inventory; HAMD = Hamilton Depression Scale, RR= blood pressure, BMI= Body mass index.

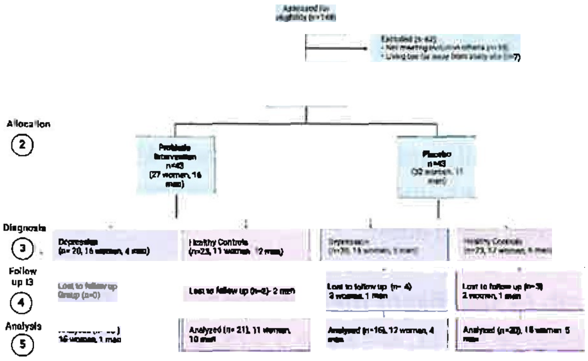

Figure 1. Consort Flow Diagram

HRV

24-hour HRV measurements

The repeated measures ANOVA for the 24-hour mean heart rate (meanHR) revealed a significant interaction between time and probiotics, F(3, 198) = 3.244, p = .027, q^2 = 0.047, as well as between time and diagnosis, F(3, 195) = 4.351, p = .021, q' = 0.050. Depressed patients showed significantly higher heart rates compared to healthy controls (F(1, 66) = 18.048, p < .001) (see Figure 3).
For pNN50, RMSSD and SDNN over 24 hours, there was a significant effect of diagnosis (F(1, 65) = 6.871, p = 0.011; F(1, 66) = 9.937, p = 0.002; and F(1, 66) = 14.257, p < 0.001, respectively), with patients with depression having significantly lower values than healthy controls. However, there were no significant differences between the probiotic and placebo groups and across time points. Furthermore, there were no significant differences in terms of lnHF. With regard to 24-hour logRSA, the repeated measures ANOVA showed a significant interaction between time and diagnosis (F (3, 198) = 2.791, p = 0.042, q' = 0.041).

Morning and afternoon HRV measurements

In the repeated measures ANOVA, morning heart rate showed a significant interaction of time and probiotic intake (F(3, 192) = 3.987, p = .009, $q^{(2)}$ = 0.059) and an interaction of *time-probiotic diagnosis (F(*3, 192) = 6.062, p < .001, q(2) = 0.087). Depressed patients who took a probiotic had significantly lower heart rates after 3 months compared to those who took a placebo. There was also a significant difference between participants with depression and healthy controls ((F(1, 64) = 9.722, p = 0.003)), with patients showing significantly higher heart rates compared to healthy controls. For afternoon values, heart rate showed no significant interactions for time, but the significant between-subjects effect of diagnosis remained ((F(1, 64) = 11.723, p = 0.001)).

RMSSD in the morning showed a significant interaction effect of timeProbioticsoiagnosis, F(3, 192) = 4.181, p = .007, q^2= 0.061; as well as a significant main effect of diagnosis, with depressed patients showing lower RMSSD values compared to healthy controls (F(1, 64) = 9.919, p = 0.004). For the afternoon, only the significant difference between diagnoses remained ((F(1, 66) = 7.795, p = 0.007)).

In the repeated measures ANOVA, InHF showed a significant interaction of timeProbioticDiagnosis in the morning (F(2,726, 192) = 2.986, p = 0.037, q' = 0.016). There was also a significant difference between participants in terms of diagnosis ((F(1, 64) = 6.800, p = 0.011)). For the afternoon, only the significant difference between diagnoses remained ((F(1, 66) = 6.394, p = 0.014)

For logRSA in the morning, there was a significant interaction effect of time*probiotics and diagnosis, F(3, 192) = 3.744, p = 0.012, q' = 0.055). Similarly, there was a significant difference in IogRSA between patients with depression and healthy controls (F(1, 64) =8.799, p=0.004). For the afternoon, only the significant difference between diagnoses remained (F(1, 66) = 8.860, p = 0.004).

In terms of heart rate, there was only one significant main effect for diagnosis, with participants with depression having a higher heart rate during quiet sleep ($F(1, 68) = 16.479$, $p = 0.001$) and restless sleep ($F(1, 68) = 13.402$, $p = 0.001$).

For pNN50, a significant difference was found only between patients with depression and healthy controls during quiet sleep ($F(1, 68) = 8.301$, $p = 0.006$) and during restless sleep ($F(1, 68) = 8.204$, $p = 0.006$).

For RMSSD during quiet sleep, there was a significant interaction of time and diagnosis ($F(3, 204) = 3.075$, $p = 0.029$, $q^* = 0.043$) and a significant difference between patients with depression and healthy controls ($F(1, 68) = 6.753$, $p = 0.011$), while in restless sleep there was only a significant difference between patients and controls ($F(1, 68) = 9.155$, $p = 0.003$).

There was also a significant difference between patients and controls in the HF measurements during quiet and restless sleep (quiet sleep: $F(1, 68) = 6.919$, $p = 0.011$; restless sleep: $F(1, 68) = 7.787$, $p = 0.007$).

With regard to logRSA, significant differences between patients and controls were also found in restless sleep ($F(1, 68) = 8.204$, $p = 0.006$) and quiet sleep ($F(1, 68) = 8.031$, $p = 0.006$).

For SDNN in quiet sleep, there was a significant interaction of time*diagnosis ($F(3, 204) = 2.777$, $p = 0.049$, $q' = 0.039$) and a significant difference between patients and controls ($F(1, 68) = 7.172$, $p = 0.009$). During restless sleep, there was only a significant difference in terms of diagnosis ($F(1, 68) = 12.336$, $p < 0.001$).

Microbiome

A total of 313 stool samples were available (143 samples from patients with depression and 170 samples from healthy controls, 157 samples from participants who received probiotics and 156 samples from participants who received a placebo), with samples from 66 of 86 participants (76.74%) available for all time points.

In terms of Chao-1 diversity ($F(1, 62) = 6.094$, $p = 0.017$), number of observed species ($F(1, 62) = 7.865$, $p = 0.007$), Simpson index ($F(1, 62) = 5.119$, $p = 0.027$) and Shannon index ($F(1, 62) = 9.555$, $p = 0.003$), there was a significant between-subject effect for diagnosis, with depressed patients exhibiting lower alpha diversity. However, alpha diversity was not significantly altered by probiotic intake or over time.
There was a significant difference in beta diversity at baseline between patients with depression and healthy controls ($p = 0.001$). PCoA and redundancy analysis showed no significant effect of probiotics on the overall composition of the microbiome over time.

Further parameters relating to questionnaires and laboratory parameters are currently still being evaluated.

Conclusions:

The present study provides valuable insights into the potential mechanisms of action of probiotics in major depression (MD). The results suggest that daily intake of a multi-strain probiotic over a period of three months can significantly improve morning vagal function in patients with MD. This improvement could be due to increased vagal activation, as observed in animal studies, and supports the hypothesis that probiotics may mediate antidepressant effects by stimulating the vagus nerve.

Although the composition of the gut microbiome differed between depressed patients and healthy controls at the start of the study, the probiotic intervention did not have a significant effect on the overall diversity of the microbiome.
Interestingly, however, specific changes in the microbiome were observed, notably an increase in Christensellales and a decrease in Ruminococcus in the depressed participants after three months of probiotic treatment.

These findings suggest that probiotics may represent potential therapeutic options for the treatment of MD by targeting the physiological interactions between the gut and the brain. Future research should further investigate the exact mechanisms and conduct larger, randomised controlled trials to better understand the long-term effects and clinical relevance of these interventions.
