# Supplementary material for: Do probiotics modulate dietary intake? Pilot data from a randomized controlled sub-study of the ProBioHRV clinical trial in patients with depression and healthy controls
Source: PLoS One. 2026 Jun 23;21(6):e0350801. doi: 10.1371/journal.pone.0350801 (PMC13289889; doi:10.1371/journal.pone.0350801)
Supplement: S4 File — (PDF) [file pone.0350801.s004.pdf]

Study protocol version 1.2 dated 3 July 2021

Pilot study: Probiotics and the gut-brain axis – Do probiotics interact with the vagus nerve?

Pilot study: Probiotics and the gut-brain axis – Do probiotics interact with the vagal nerve?

ProBIO-HRV study

Applicant: Priv. Doz. DDr. Sabrina Mörkl Medical

University of Graz
University Clinic for Psychiatry and Psychotherapeutic Medicine

Medical treatment and study team:
Prof. Dr Eva Reininghaus, Dr Susanne Bengesser, Dr Jolana Wagner-Skacel, Dr Adelina Tmava, Dr Katja Großschädl,
Dr. Frederike Fellendorf, Dr. Rene Pilz, Dr. Melanie Schweinzer
***Blood processing*:**
Renate Unterweger (MTA), Priv. Doz. Andreas Meinitzer
Collaborations with MedUni Graz:
Associate Professor Priv.-Doz.Mag. Dr. Sandra Holasek, University Professor Dr. Harald Mangge, Priv. Doz.
Dr Andreas Meinitzer,
Prof. Christine Moissl-Eichinger, Prof. Nandu Goswami, Dr Bianca Brix, Ruslan Neshev
Company collaboration:
Allergosan

Abstract

Justification:

Depression is one of the most common and serious illnesses worldwide and has a significant negative impact on patients' quality of life and ability to work. Patients with depression exhibit changes in the gut microbiome and its metabolites, which in turn influence the gut-brain axis as a bidirectional communication system. A key component of this axis is the vagus nerve, which transmits information from the internal organs in the periphery to the central nervous system. Studies have shown that the function of the vagus nerve is impaired in patients with depression. At the same time, depressed patients often have a pro-inflammatory status, which is linked to the function of the vagus nerve in influencing the immune response. Animal models have shown that vagal afferents transmit information from intestinal bacteria directly to the central nervous system and can thus induce both anxious and depressive behaviour. Stimulation of the vagus nerve (which is currently used clinically as an invasive procedure for treatment-resistant depression) has been shown to improve depression and alleviate (neuro)inflammation. A pilot study from our working group has shown that the diversity of the gut microbiome in women correlates with the function of the vagus nerve (Mörkl/Oberascher et al., 2020, in prep).

Relevance: To date, no research has been conducted into whether and to what extent probiotics can influence the function of the vagus nerve. This new mechanism could be used to specifically alter the function of the vagus nerve with probiotics and provide a rationale for add-on therapy with probiotics ("psychobiotics").

Design: This monocentric study will investigate the effect of a multi-strain probiotic on vagus nerve function in 40 depressed patients and 40 healthy control subjects in a placebo-controlled setting. The study participants will receive either a probiotic or a placebo for 3 months, and the function of the vagus nerve will be determined at 4 time points using a portable ECG, which will be worn for 24 hours. In addition, inflammation markers (hs-CRP, IL-6) and the gut microbiome were determined using 16S sequencing. This is the first study worldwide to investigate the effects of a multistrain probiotic on the vagus nerve in depressed patients compared to healthy control subjects. The results of this study could contribute to a better understanding of the basis of probiotic effects on mental processes.

.

Content

1. Scientific background 4

Previously available data 6

2. Objectives of the study 6

3. Study procedure 7

3.1 Inclusion and exclusion criteria 8

3.2 Questions and hypotheses 9

4. Target values 10

4.1 Main target values 10

4.2 Secondary targets 10

5. Methods 10

5.1 Intervention with multi-species probiotic 10

5.2 Questionnaires 11

5.3 Heart rate variability (HRV) 11

6. Statistics 13

6.1 Randomisation 13

6.1 Data analysis and evaluation 14

6.2 Considerations regarding case numbers 15

6.3 Microbiome statistics 15

1. Scientific background

According to the WHO, depression is one of the most common diseases, and the associated health and socio-economic problems place a heavy burden on both those affected and society as a whole. Despite intensive efforts to improve the treatment of depression, psychopharmacological therapy still only leads to complete remission in one third of patients (1). Combination therapies of antidepressants and psychotherapy are used to treat depression, with approximately 50% of patients discontinuing antidepressant treatment prematurely due to side effects (2). This underscores the need for further research into the mechanisms underlying this disease in order to develop more tolerable and effective treatment options.

Depressed patients predominantly show profound changes in their gut microbiota composition, which in turn affects the intestinal barrier and inflammatory status. The largest study currently available on the gut microbiome and depressive symptoms has shown that while the butyrate-producing *Faecalibacterium* and Coprococcus bacteria are associated with a higher quality of life, *Dialister* and *Coprococcus* spp. are negatively correlated with depression (3). A meta-analysis of intervention studies with probiotics showed a significant improvement in depressive mood compared to healthy controls (SMD = -1.62, 95% CI = -2.73 to -0.51, $p< 0.01$) (4). However, the exact mechanisms responsible for improving depression are still the subject of research.

In our pilot study, we were able to show that the diversity of the gut microbiota correlates with the function of the vagus nerve, the tenth cranial nerve and main player in the microbiota-gut-brain axis (Oberascher/Mörkl et al., 2020, in prep.). This points to an interaction between the gut microbiome, inflammation and the function of the vagus nerve. The afferents of the vagus nerve are polymodal and therefore respond to mechanical, chemical and hormonal signals (5, 6). Stress and depression influence its function. Depressed patients show reduced vagus function and reduced heart rate variability, which is a biomarker of the vagus nerve (7, 8). In addition, there is increasing

evidence that the nervous system controls inflammatory processes and dampens the immune response via the vagus nerve and oxytocin (9), with heart rate variability (HRV) being particularly associated with blood levels of C-reactive protein (CRP) (10) and interleukin-6 (IL-6). The inflammatory hypothesis of depression describes how inflammatory processes in the brain lead to depressed mood and anxiety (11, 12). Older reports on vagotomy in humans, which was previously performed to treat peptic ulcer disease, show a positive correlation with the occurrence of psychiatric disorders (13, 14). and underscore the important role of the vagus nerve in the pathogenesis of depression. In addition, experimental procedures for electrical vagus nerve stimulation are now being used to treat treatment-resistant depression (15).

Our previous studies suggest that changes in the gut microbiome are associated with psychiatric disorders (16, 17) and that probiotic interventions affect the inflammatory status (IL-6) in depressed patients by reducing interleukin-6 gene expression (18) and that gut microbiota diversity correlates with vagus nerve function as measured via HRV (logarithm of respiratory sinus arrhythmia (logRSA), HR, PRQ) (Oberascher/Mörkl et al., 2021, in prep.). Probiotics alter the composition of gut bacteria and their metabolites (18) and could thus influence vagal afferents that transmit signals to the hypothalamus via the nucleus tractus solitarius (NTS). In animal models, bacteria such as *Escherichia coli* were able to trigger vagal afferent neurons and act on the CNS (19). Gut bacteria such as *Bifidobacterium longum* use the vagus nerve to transmit signals from the gastrointestinal tract to the brain (20, 21).

Heart rate variability (HRV) measures cardiac autonomic regulation and describes the degree of fluctuation in heartbeats (R-R intervals) that can be derived from an electrocardiogram. These intervals are not constant and vary with each heartbeat. Basically, a higher HRV means better vagus nerve function and better health (10). Reduced vagus nerve function is associated with a reduction in HRV and a monotonous regularity of heart rate (22). A meta-analysis of HRV in depressed patients showed that they had reduced HRV indices compared to healthy control subjects and that depression correlated negatively with HRV (23). HRV is associated with inflammatory markers (CRP and IL-6). For example, Jarczok et al. showed that vagus nerve function could predict CRP levels (10, 23).

Data available to date

Studies conducted by our working group indicate changes in the gut microbiome in mental illnesses such as depression, bipolar disorder and anorexia nervosa (16, 17, 24). In addition, we were able to show that the administration of a multi-species probiotic significantly influences interleukin-6 gene expression in depressed patients compared to placebo (18). To the best of our knowledge, however, there are only a few studies that have investigated the influence of probiotics on vagus nerve function, and no psychiatric studies on this topic, although studies in mice have shown promising effects of probiotics on vagus nerve function (25). There is also an interventional study with a multispecies probiotic in women with hypertension. Romao da Silva et al. observed changes in HRV after an 8-week intervention with *Lactobacillus paracasei LPC-37, Lactobacillus rhamnosus HN001, Lactobacillus acidophilus NCFM, and Bifidobacterium lactis HN019 (*10^9CFUs for each strain), indicating an improvement in autonomic regulation. This mechanism has not yet been researched in relation to mental illness.

2. Aims of the study

The aim of this study is to investigate the effects of probiotics on vagus nerve function in patients with depression (mild, moderate and severe depressive episodes, or recurrent depressive disorder). Furthermore, the 3-month administration of a multi-species probiotic will be used to evaluate a possible improvement in depressive symptoms. The change should be measurable not only clinically, but also on the basis of various markers (i.e. in the areas of inflammation, tryptophan metabolism and the microbiome). For this pilot study, we will invite patients who are being treated for depression as inpatients at the University Clinic for Psychiatry and Psychotherapeutic Medicine.

The clinical examinations will be carried out by the above-mentioned study team (i.e. using MedUni's own funds), but a study nurse/study assistant will be required to coordinate the visits for the duration of the study. The biological parameters will be evaluated by cooperation research partners (Graz). A

funding application has been sent to the City of Graz. Part of the costs will also be financed by Allergosan. The probiotics and placebo will be provided by Allergosan.

3. Study procedure

Study design

This study is being conducted at the University Clinic for Psychiatry and Psychotherapeutic Medicine as a monocentric, randomised study. Randomisation is carried out by Allergosan (block randomisation). Patients at the University Clinic for Psychiatry who meet the inclusion criteria are invited to participate. Healthy control subjects will be recruited by Probando (probando.io) as well as via social media and notices on bulletin boards. A total of four **study visits** will take place. During the clinical visits, blood samples will be taken, psychological and cognitive tests will be conducted, and a clinical interview will be held to record any possible side effects and symptoms. The psychiatric diagnosis is made at the start of the study by a psychiatrist using the
M.I.N.I. (Mini International Neuropsychiatric Interview [24].

Study visits

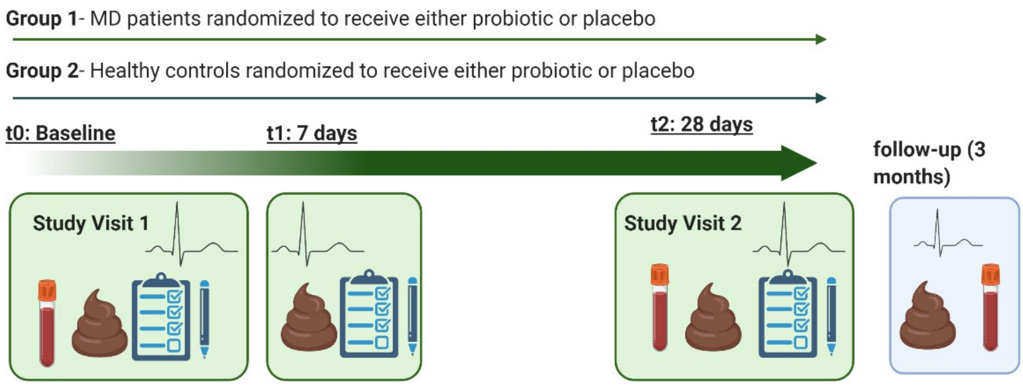

Figure 1. Study visits

Visit 1 Procedure:

- Blood sampling

- Stool
- Heart rate variability (device is attached and worn for 24 hours)
- Each patient receives a month's supply of probiotics (approx. 30 capsules in total) labelled with their assigned number. The first dose is prepared and consumed together with the study doctor or study psychologist.
- Anthropometry
- Questionnaires

Visit 2 Procedure:
- Patient compliance is measured by having patients bring their daily probiotic rations back to the visit (i.e. after 1 month) and checking and noting the number of doses taken or not taken.
- Blood
- Stool (can be brought along, max. 1 day old and stored in a cool place) including stool log
- Anthropometry
- Questionnaires
- Heart rate variability (device is attached and worn for 24 hours)
- Each patient receives a ration of probiotics labelled with the respective assigned number for 2 additional months (a total of approx. 60 units).

Follow-up appointment (i.e. after 3 months):
- Blood sample
- Stool (can be brought along, max. 1 day old and stored in a cool place) including stool log
- Anthropometry
- Questionnaires
- Heart rate variability (device is attached and worn for 24 hours)

3.1 Inclusion and exclusion criteria

Inclusion criteria for this study are: informed consent, diagnosis of depression according to ICD-10, age between 18 and 65 years.

Exclusion criteria are: suicidal tendencies, lack of consent or capacity to consent, known cardiovascular disease, pregnancy, breastfeeding, pronounced dependence on alcohol or psychotropic substances (benzodiazepines, morphine), other severe mental or organic diseases (epilepsy, brain tumours, trauma, recent major surgery), tumour diseases, dementia (Mini Mental Score

<20), severe autoimmune diseases or immunosuppression (lupus erythematosus, HIV, multiple sclerosis), antibiotic therapy in the last month, laxative abuse, acute

infections, diarrhoea, gastrointestinal surgery (except appendectomy). Participants should not have taken probiotics in the last 6 months. Furthermore, they should not consume any additional dietary supplements or probiotics during the study period. The additional intake of antibiotics or prebiotic supplements is also considered an exclusion criterion.

3.2 Questions and hypotheses

The **hypotheses** of this study are as follows:

Primary hypotheses:

H(0):

A 4-week intake of a multi-species probiotic shows no significant increase in vagus function (measured by HRV, log RSA) and no significant decrease in inflammation parameters (IL-6, hsCRP) in subjects with depression and in healthy control subjects compared to placebo treatment.

H1:
A 4-week intake of a multi-species probiotic shows a significant increase in vagal function (measured by HRV, log RSA) and a significant decrease in inflammation parameters (IL-6, hsCRP) in subjects with depression and in healthy control subjects compared to placebo treatment.

Secondary hypotheses:

1) Depressed patients show significantly higher inflammatory parameters and lower vagus function (logRSA) at the start of the study compared to healthy control subjects.
2) The vagus function (logRSA) and inflammatory parameters (hsCRP, IL-6) of depressed patients and healthy control subjects do not differ significantly after 3 months of intervention.
3) Vagus function correlates with depression scores, inflammatory parameters, oxytocin, and gut microbiota (in diversity, composition, and function) in both healthy control subjects and depressed patients.

4) Stool samples from the intervention group show a significantly higher proportion of short-chain fatty acids after the intervention.
5) The content of short-chain fatty acids in stool correlates with vagus function in healthy control subjects and in depressed patients.
6) Vagus nerve function correlates negatively with depression scores, sleep quality scores, subjective stress experience, and the diversity and composition of the gut microbiome.

4. Target parameters

4.1 Primary endpoints

The target parameters for the main hypothesis are logRSA of HRV as a marker of vagus nerve function and the inflammation markers (hs-CRP, IL-6).

4.2 Secondary endpoints

Blood parameters: oxidative stress markers, tryptophan metabolism, serum oxytocin, TNF-alpha, INF, IGF, neurotrophins (e.g. BDNF), salivary amylase; gut microbiome, metabolome, questionnaire scores, height, weight, BMI, gender, age, smoking habits, medication.

5. Methods

5.1 Intervention with multispecies probiotic

The multistrain probiotic and placebo used are provided by the Allergosan Institute. This is a commercially available dietary supplement called OMNi-BiOTiC® -SR, which contains 9 bacterial strains (*Bifidobacterium bifidum W23, Bifidobacterium lactis W51, Bifidobacterium lactis W52, Lactobacillus acidophilus W22, Lactobacillus casei W56, Lactobacillus paracasei W20, Lactobacillus plantarum W62, Lactobacillus salivarius W24, Lactococcus lactis W19)* with at least 7.5 trillion microorganisms per serving (=3g).

The placebo preparation will have the same colour, consistency and taste as the probiotic product. The matrix of the product contains corn starch, maltodextrin, inulin, potassium chloride, magnesium sulphate, fructooligosaccharides, amylases and manganese sulphate. Patients and healthy controls receive OMNi-Biotic SR after it has been dissolved in water and the activation time of 10 minutes has been observed. For inpatients, the study preparation is prepared daily at 7:00 a.m. by study staff and given to inpatients before breakfast and in the evening every day until discharge. After discharge, patients continue to take the preparation twice daily at home on their own. The entire study team is blinded.

5.2 Questionnaires

To verify the diagnosis, a M.I.N.I. is performed on depressed patients who have been admitted to the University Clinic for Psychiatry and Psychotherapeutic Medicine.

M.I.N.I. is administered. The severity of depressive symptoms is assessed using the **Hamilton Scale for Depression (HAMD)** and the **Beck Depression Inventory (BDI)**. In addition, sleep quality is assessed using the **Pittsburgh Sleep Quality Inventory (PSQI)**. Cognitive reactivity to depressive mood is assessed using the **Leids-R questionnaire**. Stress is measured using the **Trier Inventory of Chronic Stress (TICS)**. In addition, the **Perceived Stress Scale** is used to assess recent stress levels. We measure attachment behaviour using the **Adult Attachment Scale.** We also record the following clinical and demographic parameters: height, weight, BMI, gender, age, smoking habits, medication. Nutritional history is assessed using **the Vienna Nutrition Protocol (**24-hour recall), and physical **activity** history is assessed using **the International Physical Activity Questionnaire.** Completing these questionnaires takes approximately 1-1.5 hours.

5.3 Heart rate variability (HRV)

The portable ECG monitor "*eMotion* Faros" (http://ecg.biomation.com/faros.htm) is used to record heart rate variability. The small and portable ECG device is attached to the upper body of the participants and worn for 24 hours at the time of testing. The device is easy to use and is already being used successfully in several areas such as occupational medicine, cardiology, research, physiotherapy, sports counselling and lifestyle counselling.

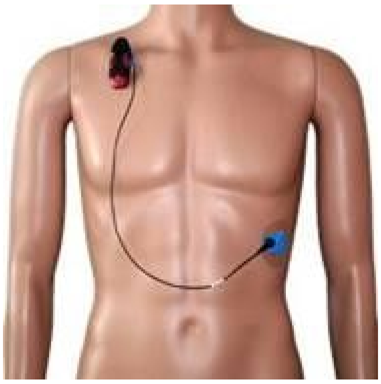

Figure 2: ECG device for recording heart rate variability. Technical details: ECG sampling up to 250 Hz (adjustable); HRV: 1000 Hz sampling; 3D acceleration (activity): sampling up to 25 Hz (adjustable).

HRV analysis will be performed according to the Task Force guidelines (8). The following parameters are calculated to measure autonomic regulation: standard deviation of all normal-to-normal (NN) intervals (SDNN); log-transformed value of respiratory sinus arrhythmia (logRSA); the natural logarithm (ln) of high frequency (lnHF) and low frequency (lnLF) band; ln of total spectral power (lnTOT); LF/HF ratio, pulse-rate-to-respiratory-rate ratio (PRQ); and sleep duration.

5.4 Microbiome analysis

One gram of stool is collected at three test times and immediately stored in an -80°C freezer for further analysis. The microbiome is evaluated using Illumina MiSeq according to a previously published procedure (Klymiuk et al., 2017) [32]. FastQ files are used for data analysis. Quantitative Insights Into Microbial Ecology (QIIME II) is used on the MedUni Graz galaxy server to evaluate the gut microbiome (galaxy.medunigraz.at). Depending on financial resources, the metabolome (from stool) will also be analysed.

5.5 Blood sampling

Blood samples are taken from all participants in the morning on an empty stomach. A maximum of 70 ml of blood is taken in total. After blood collection, the plasma is centrifuged at 4000 rpm for 15 minutes and stored at -20 °C for further analysis. C-reactive protein (CRP) and (IL)-6 are analysed at the Institute for Laboratory Diagnostics (Prof. Mangge, Doz. Meinitzer) using Cobas 6000 (Roche Diagnostics, Mannheim, Germany).

The following parameters are also determined:

Routine parameters: blood count including differential blood count, liver values, kidney values, lipids, CRP, IL-6, glucose metabolism
Biomarkers: oxidative stress parameters, oxytocin, neuroinflammatory markers (e.g. interleukins, TNF a, INF, tryptophan metabolism, IGF), neurotrophins (e.g. BDNF), depending on the available budget from funding, the samples are analysed immediately or stored in the meantime.

5.6 Saliva sample

At the time of inclusion in the study and after 1 and 4 weeks, alpha-amylase in saliva will be measured as a stress marker, provided sufficient funding can be secured. Salivary amylase is elevated as a stress marker in depressed patients (26). Studies have shown that probiotics can influence alpha-amylase in athletes or surgical patients (27, 28) – whether probiotics can influence salivary amylase in depressed patients has not yet been investigated. A saliva sample is required to determine alpha-amylase. It is obtained non-invasively by the participants themselves using a Salivette (Sarstedt, Germany) in accordance with the test instructions (on an empty stomach, without brushing their teeth or using mouthwash beforehand), then centrifuged and frozen until analysis. The evaluation will be carried out in collaboration with the Otto Loewi Research Centre for Pathophysiology and Immunology.

6. Statistics

6.1 Randomisation

The study design results in 4 groups:

- **Depressed patients Probiotics/placebo**
- **Healthy subjects: probiotics/placebo**

The subjects are using computer-assisted **4-person block randomisation** (randomisation.com).

6.1 Data analysis and evaluation

The data obtained will be analysed using IBM SPSS, version 27. A descriptive data description (mean, standard deviation, percentile rank) will be provided. Correlations between variables will be calculated using Pearson's correlation coefficient or Spearman's correlation coefficient, depending on the distribution. Error probabilities below $p < 0.5$ are considered statistically significant. Microbiome analyses are performed in R (version 3.6) and Rstudio (version 1.2.1555) (R-doundation, Vienna, Austria), with visualisations using the ggplot2 library.

Both an intention-to-treat and a per-protocol analysis are performed.

- **Intention-to-treat analysis:**

All inclusions (valid cases + dropouts). All parameters collected are analysed.

- **Per-protocol analysis:**

All inclusions without meeting a drop-out criterion (all valid cases). All parameters collected are analysed. The per-protocol analysis is the primary evaluation concept.

Dropout criteria:

The following events/situations lead to the affected case being classified as a dropout and thus not being included in the per-protocol analysis:

- Serious violations of the project plan
- (With the exception of the test product:) Intake/consumption of (relevant amounts of) probiotics
- Incorrect inclusion
- Failure to follow up
- Unavailability of data required for the calculation of the primary endpoint parameters
- Unblinding
- Withdrawal of consent to participate in the study by the patient

6.2 Considerations regarding the number of cases

This project is a pilot study. To the best of our knowledge, probiotic effects on vagus function in depressed patients have never been investigated before. A power analysis was performed using G*Power 3.1.(29). In the F-tests category, the

"Repeated Measures ANOVA within-between interaction" was selected as the procedure. With a low effect size of 0.2, alpha 0.05 and 95% power, a total of 80 participants (20 participants per group) is required for 4 groups (healthy subjects: probiotic/placebo; depressed patients: probiotic/placebo).

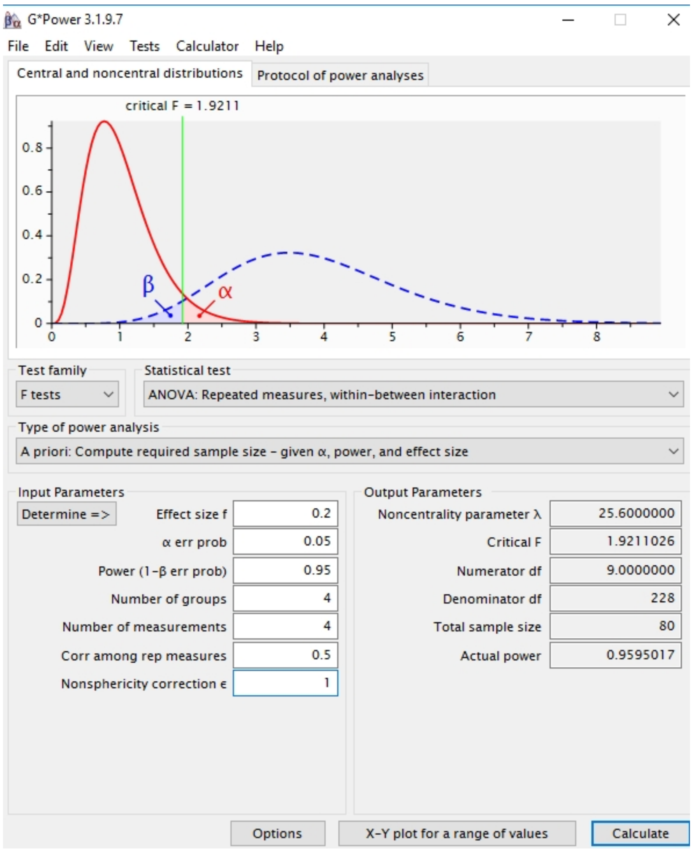

Figure 3: Sample size calculation with G*Power

6.3 Microbiome statistics

Microbiome statistics are performed using R (v3.6) and Rstudio (1.2.1555) (R-foundation, Vienna, Austria) in collaboration with our cooperation partner Dr Thomaz Bastiaanssen, APC Microbiome Institute Cork. The data is visualised using the ggplot2 library. To compare the alpha diversity between the intervention and placebo groups, the Chao-1 diversity index, the Simpson index and the Shannon index are used

The diversity indices are calculated using the iNEXT library [35]. CoDa is used due to the compositional nature of microbiome data (Gloor, et al., 2017). Beta diversity is measured using principal component analysis (PCA). To correct for multiple testing, Storey's q-value post-hoc procedure is used with a q-value of 0.1 as the cut-off (30). Piphillin is used for functional microbiome analyses (prediction of metagenomic content) (31).

References

1. Klesse C, Berger M, Bermejo I, Bschor T, Gensichen J, Harfst T, et al. Evidence-based psychotherapy for depression. Psychotherapeut. 2010;55(3):247-63.
2. Sansone RA, Sansone LA. Antidepressant adherence: are patients taking their medications? Innovations in clinical neuroscience. 2012;9(5-6):41.
3. Valles-Colomer M, Falony G, Darzi Y, Tigchelaar EF, Wang J, Tito RY, et al. The neuroactive potential of the human gut microbiota in quality of life and depression. 2019;4(4):623-32.
4. Sanada K, Nakajima S, Kurokawa S, Barceló-Soler A, Ikuse D, Hirata A, et al. Gut microbiota and major depressive disorder: A systematic review and meta-analysis. 2020;266:1-13.
5. Berthoud HR, Blackshaw LA, Brookes SJ, Grundy D. Neuroanatomy of extrinsic afferents supplying the gastrointestinal tract. Neurogastroenterol Motil. 2004;16 Suppl 1:28-33.
6. Egerod KL, Petersen N, Timshel PN, Rekling JC, Wang Y, Liu Q, et al. Profiling of G protein-coupled receptors in vagal afferents reveals novel gut-to-brain sensing mechanisms. Mol Metab. 2018;12:62-75.
7. Sgoifo A, Carnevali L, Pico Alfonso MdlA, Amore MJS. Autonomic dysfunction and heart rate variability in depression. 2015;18(3):343-52.
8. Bassett DJA, Psychiatry NZJo. A literature review of heart rate variability in depressive and bipolar disorders. 2016;50(6):511-9.
9. Matteoli G, Boeckxstaens GE. The vagal innervation of the gut and immune homeostasis. Gut. 2013;62(8):1214-22.
10. Jarczok MN, Kleber ME, Koenig J, Loerbroks A, Herr RM, Hoffmann K, et al. Investigating the associations of self-rated health: heart rate variability is more strongly associated than inflammatory and other frequently used biomarkers in a cross sectional occupational sample. 2015;10(2):e0117196.
11. Scott D, Happell B. The high prevalence of poor physical health and unhealthy lifestyle behaviours in individuals with severe mental illness. Issues Ment Health Nurs. 2011;32(9):589-97.
12. Teasdale SB, Ward PB, Samaras K, Firth J, Stubbs B, Tripodi E, et al. Dietary intake of people with severe mental illness: systematic review and meta-analysis. British Journal of Psychiatry. 2019;214(5):251-9.
13. Browning JS, Houseworth JH. Development of new symptoms following medical and surgical treatment for duodenal ulcer. Psychosom Med. 1953;15(4):328-36.
14. Whitlock FA. Some Psychiatric Consequences of Gastrectomy. BMJ-Brit Med J. 1961;1(523):1560-+.
15. Groves DA, Brown VJJN, Reviews B. Vagal nerve stimulation: a review of its

applications and potential mechanisms that mediate its clinical effects. 2005;29(3):493-500.
16. Mörkl S, Lackner S, Meinitzer A, Mangge H, Lehofer M, Halwachs B, et al. Gut microbiota, dietary intakes and intestinal permeability reflected by serum zonulin in women. 2018;57(8):2985-97.
17. Painold A, Mörkl S, Kashofer K, Halwachs B, Dalkner N, Bengesser S, et al. A step ahead: Exploring the gut microbiota in inpatients with bipolar disorder during a depressive episode. Bipolar Disord. 2019;21(1):40-9.
18. Reiter A, Bengesser SA, Hauschild A-C, Birkl-Töglhofer A-M, Fellendorf FT, Platzer M, et al. Interleukin-6 Gene Expression Changes after a 4-Week Intake of a Multispecies Probiotic in Major Depressive Disorder—Preliminary Results of the PROVIT Study. 2020;12(9):2575.
19. Gakis G, Mueller M, Hahn J, Glatzle J, Grundy D, Kreis MJAN. Neuronal activation in the nucleus of the solitary tract following jejunal lipopolysaccharide in the rat. 2009;148(1-2):63-8.
20. Goehler LE, Gaykema RP, Opitz N, Reddaway R, Badr N, Lyte M. Activation in vagal afferents and central autonomic pathways: early responses to intestinal infection with Campylobacter jejuni. Brain Behav Immun. 2005;19(4):334-44.
21. Bercik P, Park A, Sinclair D, Khoshdel A, Lu J, Huang X, et al. The anxiolytic effect of Bifidobacterium longum NCC3001 involves vagal pathways for gut–brain communication. Neurogastroenterology & Motility. 2011;23(12):1132-9.
22. Leistedt SJ, Linkowski P, Lanquart JP, Mietus J, Davis RB, Goldberger AL, et al. Decreased neuroautonomic complexity in men during an acute major depressive episode: analysis of heart rate dynamics. 2011;1(7):e27-e.
23. Kemp AH, Quintana DS, Gray MA, Felmingham KL, Brown K, Gatt JMJBp. Impact of depression and antidepressant treatment on heart rate variability: a review and meta-analysis. 2010;67(11):1067-74.
24. Mörkl S, Lackner S, Muller W, Gorkiewicz G, Kashofer K, Oberascher A, et al. Gut microbiota and body composition in anorexia nervosa inpatients in comparison to athletes, overweight, obese, and normal weight controls. Int J Eat Disord. 2017;50(12):1421-31.
25. Tunapong W, Apaijai N, Yasom S, Tanajak P, Wanchai K, Chunchai T, et al. Chronic treatment with prebiotics, probiotics and synbiotics attenuated cardiac dysfunction by improving cardiac mitochondrial dysfunction in male obese insulin-resistant rats. 2018;57(6):2091-104.
26. Bauduin SEEC, van Noorden MS, van der Werff SJA, de Leeuw M, van Hemert AM, van der Wee NJA, et al. Elevated salivary alpha-amylase levels at awakening in patients with depression. Psychoneuroendocrinology. 2018;97:69-77.
27. Kurdi M, Ramaswamy A, Kumar L, Choukimath S, Jangi A. Use of a non-invasive biomarker salivary alpha-amylase to assess the role of probiotics in sleep regulation and stress attenuation in surgical patients: A randomised double-blind clinical trial. Indian Journal of Anaesthesia. 2021;65(5):390-7.
28. Pumpa KL, McKune AJ, Harnett J. A novel role of probiotics in improving host defence of elite rugby union athletes: A double-blind randomised controlled trial. J Sci Med Sport. 2019;22(8):876-81.
29. Faul F, Erdfelder E, Lang A-G, Buchner A. G* Power 3: A flexible statistical power analysis programme for the social, behavioural, and biomedical sciences. Behaviour research methods. 2007;39(2):175-91.
30. Storey JD, Tibshirani R. Statistical significance for genomewide studies. Proceedings of the National Academy of Sciences. 2003;100(16):9440-5.
31. Iwai S, Weinmaier T, Schmidt BL, Albertson DG, Poloso NJ, Dabbagh K, et al. Piphillin: improved prediction of metagenomic content by direct inference from human microbiomes. 2016;11(11):e0166104.
