# Supplementary material for: Do probiotics modulate dietary intake? Pilot data from a randomized controlled sub-study of the ProBioHRV clinical trial in patients with depression and healthy controls
Source: PLoS One. 2026 Jun 23;21(6):e0350801. doi: 10.1371/journal.pone.0350801 (PMC13289889; doi:10.1371/journal.pone.0350801)
Supplement: S5 File — (PDF) [file pone.0350801.s005.pdf]

Studienprotokoll Version 1.2 vom 03.07.2021

Pilotstudie: Probiotika und die Darm-Gehirn-Achse-Interagieren Probiotika mit dem Vagusnerv?

Pilot-study: Probiotics and the gut brain axis- Do probiotics interact with the vagal nerve?

ProBIO-HRV-study

Antragstellerin: Priv. Doz. DDr. Sabrina Mörkl

Medizinische Universität Graz
Universitätsklinik für Psychiatrie und Psychotherapeutische Medizin

Ärztliches Behandlungs- und Studienteam:
Prof. Dr. Eva Reininghaus, Dr. Susanne Bengesser, Dr. Jolana Wagner-Skacel, Dr. Adelina Tmava, Dr. Katja Großschädl,
Dr. Frederike Fellendorf, Dr. Rene Pilz, Dr. Melanie Schweinzer
***Blutbearbeitung*:**
Renate Unterweger (MTA), Priv. Doz. Andreas Meinitzer
Kooperationen MedUni Graz:
Assoz. Prof.Priv.-Doz.Mag. Dr. Sandra Holasek, Univ.-Prof. Dr. Harald Mangge, Priv. Doz. Dr. Andreas Meinitzer,
Univ. Prof. Dr. Christine Moissl-Eichinger, Prof. Nandu Goswami, Dr. Bianca Brix, Ruslan Neshev
Firmenkooperation:
Allergosan

Abstract

Rechtfertigung:

Depressionen zählen zu den weltweit häufigsten und schwerwiegendsten Erkrankungen und haben einen maßgeblichen negativen Einfluss auf die Lebensqualität und die Erwerbsfähigkeit der PatientInnen. PatientInnen mit Depressionen weisen Veränderungen des Darmmikrobioms und dessen Metaboliten auf, welche wiederum die Darm-Gehirn-Achse, als bidirektionales Kommunikationssystem beeinflussen. Einen Hauptbestandteil dieser Achse bildet der sogenannte Vagusnerv welcher Informationen der inneren Organe von der Peripherie ans Zentralnervensystem vermittelt. Aus Studien ist bekannt, dass die Funktion des Vagusnervs bei PatientInnen mit Depression eingeschränkt ist- parallel findet sich bei depressiven PatientInnen oft ein pro-inflammatorischer Status, der mit der Funktion des Vagusnervs die Immunantwort zu beeinflussen in Verbindung steht. Im Tiermodell konnte gezeigt werden, dass vagale Afferenzen Informationen von Darmbakterien direkt an das Zentralnervensystem vermitteln und somit sowohl ängstliches als auch depressives Verhalten induzieren können. Eine Stimulation des Vagusnervs (welche heutzutage als invasives Verfahren klinisch gegen therapieresistente Depressionen zum Einsatz kommt) kann Depressionen nachweislich verbessern und (Neuro-)inflammation lindern. Eine Pilotstudie aus unserer Arbeitsgruppe konnte zeigen, dass die Diversität des Darmmikrobioms bei Frauen mit der Funktion des Vagusnervs korreliert (Mörkl/Oberascher et al., 2020, in prep).

Relevanz: Bislang wurde noch nicht erforscht, ob und in wie weit Probiotika die Funktion des Vagusnervs beeinflussen können. Dieser neue Mechanismus könnte genützt werden um gezielt mit Probiotika die Funktion des Vagusnervs zu verändern und eine Rationale für eine Add-on Therapie mit Probiotika („*Psychobiotika*“) bilden.

Design: In dieser monozentrischen Studie soll die Wirkung eines Multistrain-Probiotikums auf die Funktion des Vagusnervs bei 40 depressiven PatientInnen und 40 gesunden Kontrollpersonen placebo-kontrolliert erforscht werden. Die StudienteilnehmerInnen bekommen über 3 Monate entweder ein Probiotikum oder ein Placebo, die Funktion des Vagusnervs wird zu 4 Zeitpunkten mittels eines tragbaren EKGs, welches für 24-h getragen wird, ermittelt. Zusätzlich werden Entzündungsmarker (hs-CRP, IL-6) und Darmmikrobiom mittels 16S Sequencing bestimmt. Dies ist die erste Studie weltweit welche die Einflüsse eines Multistrain-Probiotikums auf den Vagusnerv bei depressiven PatientInnen im Vergleich zu gesunden Kontrollpersonen untersucht. Die Ergebnisse dieser Studie könnten nachweislich dazu beitragen die Grundlagen probiotischer Wirkung auf psychische Vorgänge besser zu

verstehen.

Inhalt

1. Wissenschaftlicher Hintergrund 4
Bisher vorliegende Daten 6
2. Ziele der Studie 6
3. Studienablauf 7
3.1 Ein- und Ausschlusskriterien 8
3.2 Fragestellungen und Hypothesen 9
4. Zielgrößen 10
4.1 Hauptzielgrößen 10
4.2 Nebenzielgrößen 10
5. Methoden 10
5.1 Intervention mit Multispezies Probiotikum 10
5.2 Fragebögen 11
5.3 Herzratenvariabilität (HRV) 11
6. Statistik 13
6.1 Randomisierung 13
6.1 Datenauswertung und Evaluation 14
6.2 Überlegungen zur Fallzahl 15
6.3 Mikrobiomstatistik 15

1. Wissenschaftlicher Hintergrund

Als eine der am häufigsten auftretenden Erkrankungen laut WHO, stellen Depressioenn sowie die damit verbundenen gesundheitlichen und sozioökonomischen Probleme, für Betroffenen als auch die Gesellschaft eine große Belastung dar. Trotz intensiver Bemühungen eine Verbesserung in der Depressionsbehandlung zu erzielen, führt psychopharmakologische Therapie, auch heute, bei nur einem Drittel der PatientInnen zu vollkommener Remission (1). Für Depressionen kommen Kombinationstherapien von Antidepressiva und Psychotherapie zum Einsatz, wobei ungefähr 50% der PatientInnen vorzeitig aufgrund von Nebenwirkungen die Behandlung mit Antidepressiva abbrechen (2). Dies unterstreicht die Notwendigkeit weiterer Forschungsarbeit zu den Entstehungsmechanismen dieser Erkrankung um weitere verträgliche und wirksame Therapieoptionen zu entwickeln.

Depressive PatientInnen zeigen überwiegend tiefgreifende Veränderungen in ihrer Darm-Mikrobiotazusammensetzung, welche sich wiederum auf die Darmbarriere und den Entzündungsstatus auswirkt. In der größten derzeit verfügbaren Studie zum Darmmikrobiom und depressiven Symptomen konnte folgendes gezeigt werden: Während das Butyrat produzierende *Faecalibakterium* und die *Coprococcus*-Bakterien mit einer höheren Lebensqualität einhergehen, stehen *Dialister* und *Coprococcus* spp. in einem negativen Zusammenhang mit Depression (3). In einer Metaanalyse zu Interventionsstudien mit Probiotika konnte eine signifikante Verbesserung der depressiven Stimmungslage im Vergleich zu gesunden Kontrollen erzielt werden (SMD = -1.62, 95% CI = -2.73 to -0.51, $p< 0.01$) (4). Welche Mechanismen genau jedoch für eine Verbesserung von Depressionen verantwortlich sind, ist noch immer Gegenstand der Forschung.

In unserer Pilotstudie konnten wir darstellen, dass die Diversität der Darm-Mikrobiota mit der Funktion des Vagus-Nervs, dem zehnten Kranial-Nerv und Hauptakteur in der Mikrobiota-Darm-Gehirn-Achse korreliert (Oberascher/Mörkl et al., 2020, in prep.). Dies weist auf eine Wechselwirkung von Darm-Mikrobiom, Entzündung und der Funktion des Vagusnervs hin. Die Afferenzen des Vagusnervs sind polymodal und reagieren daher auf mechanische, chemische und hormonelle Signale (5, 6). Stress und Depression beeinflussen seine Funktion. So weisen depressive PatientInnen eine verminderte Vagusfunktion sowie eine verringerte Herzratenvariabilität, als Biomarker des Vagusnervs auf (7, 8). Zudem besteht zunehmende

Evidenz, dass das Nervensystem über den Vagus-Nerv und Oxytocin Entzündungsvorgänge steuert und die Immunantwort dämpft (9); wobei die Herzratenvariabilität (HRV) insbesondere mit Blutspiegeln des C-reaktivem Proteins (CRP)(10) und dem Interleukin-6 (IL-6) assoziiert ist. Die inflammatorische Hypothese der Depression beschreibt, wie Entzündungsprozesse im Gehirn zu gedrückter Stimmung und Ängstlichkeit führen (11, 12). Ältere Berichte zur Vagotomie beim Menschen, welche früher zur Behandlung der Ulkuskrankheit durchgeführt wurde, zeigen einen positiven Zusammenhang mit dem Auftreten psychiatrischer Krankheiten (13, 14). und unterstreichen die wichtige Rolle des Vagus-Nervs in der Pathogenese von Depressionen. Darüber hinaus werden heute experimentell Verfahren zur elektrischen Vagusnervstimulation zur Behandlung von therapieresistenten Depressionen eingesetzt (15).

Unsere vorherigen Studien legen nahe, dass Veränderungen des Darm-Mikrobioms mit psychiatrischen Störungen assoziiert sind (16, 17) und probiotische Interventionen, durch die Senkung der Interleukin-6 Genexpression, auf den Entzündungsstatus (IL-6) bei depressiven PatientInnen einwirken (18) und die Darm-Mikrobiota-Diversität mit dem der Funktion des Vagusnervs gemessen via HRV (Logarithmus der respiratorischen Sinusarrhythmie (logRSA), HR, PRQ) korreliert (Oberascher/Mörkl et al., 2021, in prep.). Probiotika verändern die Zusammensetzung der Darmbakterien und derer Metabolite (18) und könnten so vagale Afferenzen beeinflussen welche über den nucleus tractus solitarius (NTS) Signale an den Hypothalamus leiten. Im Tiermodell konnten Bakterien wie *Escherichia coli* vagale afferente Neuronen triggern und auf das ZNS wirken (19). Darmbakterien wie *Bifidobakterium longum* benützen den Vagusnerv um Signale vom Gastrointestinaltrakt zum Gehirn zu leiten (20, 21).

Die Herzratenvariabilität (HRV) misst die kardiale autonome Regulation und beschreibt den Grad der Fluktuation von Herzschlägen (R-R Intervalle) welche aus einem Elektrokardiogramm abgeleitet werden können. Diese Intervalle sind nicht konstant und bei jedem Herzschlag unterschiedlich. Grundsätzlich bedeutet eine höhere HRV eine bessere Funktion des Vagusnervs und bessere Gesundheit (10). Eine verminderte Funktion des Vagusnervs wird mit einer Verminderung der HRV und einer monotonen Regularität der Herzrate in Verbindung gebracht (22). Eine Metaanalyse zu HRV bei depressiven PatientInnen zeigte, dass diese verminderte HRV Indices im Vergleich zu gesunden Kontrollpersonen aufwiesen und depressive negativ mit HRV korrelierten (23). HRV ist mit Entzündungsmarkern (CRP und IL-6) assoziiert. Beispielsweise konnten Jarczok et al., zeigen, dass die Funktion des Vagusnervs die Höhe des CRPs vorhersagen konnte (10, 23).

Bisher vorliegende Daten

Die Studien unserer Arbeitsgruppe weisen auf Veränderungen des Darmmikrobioms bei psychischen Erkrankungen wie Depressionen, bipolarer Erkrankung und Anorexia nervosa hin (16, 17, 24). Darüber hinaus konnten wir zeigen, dass die Gabe eines Multispezies Probiotikums die interleukin-6 Genexpression bei depressiven PatientInnen im Vergleich zu Placebo signifikant beeinflusst (18). Nach unsrem besten Wissen, gibt es bislang aber nur wenige Studien welche den Einfluss von Probiotika auf die Funktion des Vagusnervs untersucht haben und noch keine psychiatrischen Studien zu diesem Thema, obwohl Studien an Mäusen vielversprechende Effekte von Probiotika auf die Funktion des Vagusnervs gezeigt haben (25). Ebenso gibt es seine interventionelle Studie mit einem Multispecies Probiotikum bei Frauen mit Hypertonie. Romao da Silva et al. konnten nach einer 8-wöchigen Intervention mit *Lactobacillus paracasei LPC-37, Lactobacillus rhamnosus HN001, Lactobacillus acidophilus NCFM, and Bifidobacterium lactis HN019* (10^9 CFUs für jeden Strain) Veränderungen der HRV feststellen, was auf eine Verbesserung der autonomen Regulation hinweist. Bei psychischen Erkrankungen wurde dieser Mechanismus bislang noch nicht erforscht.

2. Ziele der Studie

Die vorliegende Studie hat zum Ziel Auswirkungen von Probiotika auf die Funktion des Vagusnervs bei PatientInnen mit Depression (leichte, mittelgradige und schwere depressive Episode, oder rez. depressiver Störung) zu erfassen. Des Weiteren soll durch die 3-monatige Verabreichung eines Multispezies Probiotikums eine mögliche Verbesserung der depressiven Symptomatik evaluiert werden. Die Veränderung sollte nicht nur klinisch, sondern auch anhand diverser Marker messbar sein (d.h. im Bereich der Inflammation, des Tryptophanstoffwechsels, des Mikrobioms). Für diese Pilotstudie werden wir Pat., die stationär an der Univ. Klinik für Psychiatrie und psychotherapeutische Medizin aufgrund von Depressionen in Behandlung sind einladen.

Die klinischen Untersuchungen werden vom oben angeführten Studienteam durchgeführt (d.h. über MedUni Eigenmittel), allerdings wird für die Koordination der Visiten eine study nurse/Studienassistenz für die Dauer der Studie notwendig. Die Auswertungen der biologischen Parametern werden von Kooperations-Forschungspartnern übernommen (Graz). Ein

Förderungsansuchen wurde an die Stadt Graz geschickt. Ebenso wird ein Teil der Kosten von der Firma Allergosan finanziert. Die Probiotika sowie das Placebo werden von der Firma Allergosan zur Verfügung gestellt.

3. Studienablauf

Studiendesign

Diese Studie wird an der Univ. Klinik für Psychiatrie und psychotherapeutische Medizin als monozentrische, randomisierte Studie durchgeführt. Die Randomisierung erfolgt über die Firma Allergosan (Blockrandomisierung). PatientInnen der Univ. Klinik für Psychiatrie, welche die Einschlusskriterien erfüllen, werden zur Teilnahme eingeladen. Gesunde Kontrollpersonen werden von Probando (probando.io) sowie über Social Media und Aushänge an schwarzen Brettern akquiriert. Es finden insgesamt **4 Studienvisiten** statt. Zu den klinischen Visiten erfolgt eine Blutabnahme, psychologische und kognitive Tests und ein klinisches Interview um mögliche Nebenwirkungen und Symptome zu erfassen. Die psychiatrische Diagnose wird bei Studieneintritt von einem Psychiater oder einer Psychiaterin mit dem M.I.N.I. (Mini International Neuropsychiatric Interview verifiziert [24].

Studienvisiten

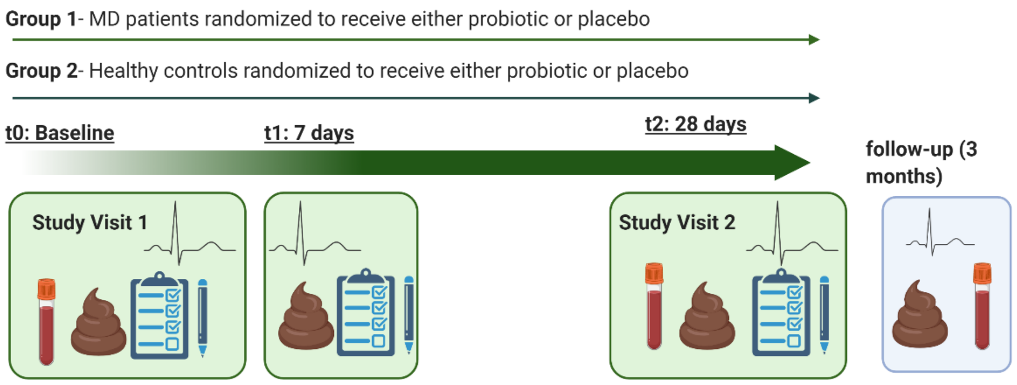

Abbildung 1. Studienvisiten

Visite 1 Ablauf:

- Blutabnahme

- Stuhl
- Herzratenvariabilität (Gerät wird angebracht und für 24 h getragen)
- Jede/r Pat. erhält die mit der jeweiligen zugeteilten Nummer versehene Ration an Probiotika für jeweils 1 Monat (insgesamt also ca. 30 Stück). Die erste Ration wird bereits gemeinsam mit dem Studienarzt/ der Studienärztin/ dem Studienpsychologen/ der Studienpsychologin zubereitet und getrunken.
- Anthropometrie
- Fragebögen

Visite 2 Ablauf:

- Die Compliance der Pat. wird gemessen, indem die Pat. ihre Probiotika-Tagesrationen zur Visite (d.h. nach 1 Monat) wieder mitbringen und die Anzahl der Einnahme bzw. Nichteinnahme geprüft und notiert wird.
- Blutabnahme
- Stuhl (kann mitgebracht werden, max. 1 Tag alt und gekühlt aufbewahrt) inklusive Stuhlprotokoll
- Anthropometrie
- Fragebögen
- Herzratenvariabilität (Gerät wird angebracht und für 24 Stunden getragen)
- Jede/r Pat. erhält die mit der jeweiligen zugeteilten Nummer versehene Ration an Probiotika für 2 weitere Monate (insgesamt also ca. 60 Stück).

Follow up Termin (d.h. nach 3 Monaten):

- Blutabnahme
- Stuhl (kann mitgebracht werden, max. 1 Tag alt und gekühlt aufbewahrt) inklusive Stuhlprotokoll
- Anthropometrie
- Fragebögen
- Herzratenvariabilität (Gerät wird angebracht und für 24 Stunden getragen)

3.1 Ein- und Ausschlusskriterien

Einschlusskriterien für diese Studie sind: Informed consent, Diagnose einer Depression nach ICD-10, Alter zwischen 18 und 65 Jahren.

Ausschlusskriterien sind: Suizidalität, mangelnde Einwilligung oder Einwilligungsfähigkeit, vorbekannte kardiovaskuläre Erkrankungen, Schwangerschaft, Stillzeit, ausgeprägte Abhängigkeit von Alkohol oder psychotropen Substanzen (Benzodiazepine, Morphine), andere schwere psychische oder organische Erkrankungen (Epilepsie, Gehirntumore, Traumata, schwere rezent stattgehabte Operationen), Tumorerkrankungen, Demenz (Mini Mental Score <20), schwere Autoimmunerkrankungen oder Immunosuppression (Lupus erythematosus, HIV, multiple Sklerose), antibiotische Therapie im letzten Monat, Laxantienabusus, akute

Infektionen, Diarrhoe, gastrointestinale Operationen (außer Appendektomie). Die Teilnehmer sollten keine Probiotika in den letzten 6 Monaten eingenommen haben, Des Weiteren sollten sie keine zusätzlichen Nahrungsergänzungsmittel, Probiotika während des Studienzeitraums konsumieren. Auch gilt die zusätzliche Einnahme von Antibiotika oder prebiotischen Supplementen als Ausschlusskriterium.

3.2 Fragestellungen und Hypothesen

Die **Hypothesen** dieser Studie sind folgende:

Primärhypothesen:

H(0):

Eine 4-wöchige Einnahme eines Multispezies Probiotikums zeigt keinen signifikanten Anstieg der Vagusfunktion (gemessen durch HRV, log RSA) und keine signifikante Abnahme der Entzündungsparameter (IL-6, hsCRP) bei ProbandInnen mit Depression und bei gesunden Kontrollpersonen im Vergleich zur Placebobehandlung.

H1:

Eine 4- wöchige Einnahme eines Multispezies Probiotikums zeigt einen signifikanten Anstieg der Vagusfunktion (gemessen durch HRV, log RSA) und eine signifikante Abnahme der Entzündungsparameter (IL-6, hsCRP) bei ProbandInnen mit Depression und bei gesunden Kontrollersonen im Vergleich zur Placebobehandlung.

Sekundärhypothesen:

1) Depressive PatientInnen zeigen signifikant höhere inflammatorische Parameter und eine niedrigere Vagusfunktion (logRSA) zu Beginn der Studie im Vergleich zu gesunden Kontrollpersonen.
2) Die Vagusfuktion (logRSA) und die inflammatorischen Parameter (hsCRP, IL-6) der depressiven PatientInnen und der gesunden Kontrollpersonen unterscheiden sich nicht signifikant nach 3 Monaten Intervention.
3) Die Vagusfunktion korreliert mit den Depressions-Scores, Entzündungsparametern, Oxytocin, Darm-Mikrobiota (in Diversität, Komposition und Funktion) sowohl bei gesunden Kontrollpersonen als auch bei depressiven PatientInnen.

4) Stuhlproben der Interventionsgruppe zeigen nach der Intervention einen signifikant höheren Anteil an kurzkettigen Fettsäuren.
5) Der Gehalt an kurzkettigen Fettsäuren im Stuhl korreliert mit der Vagusfunktion bei gesunden Kontrollpersonen und bei depressiven PatientInnen.
6) Die Funktion des Vagusnervs korreliert negativ mit Depressionsscores, den Scores für Schlafqualität, dem subjektiven Stresserleben und der Diversität und Zusammensetzung des Darmmikrobioms.

4. Zielgrößen

4.1 Hauptzielgrößen

Zielparameter für die Haupthypothese ist logRSA der HRV als Vagusfunktionsmarker und die Entzündungsmarker (hs-CRP, IL-6)

4.2 Nebenzielgrößen

Blutparameter: oxidative Stressmarker, Tryptophanstoffwechsel, Oxytocin im Serum, TNF-alpha, INF, IGF, Neurotrophine (z.B. BDNF), Speichel-Amylase; Darmmikrobiom, Metabolom, Scores der Fragebögen, Größe, Gewicht, BMI, Geschlecht, Alter, Rauchverhalten, Medikation.

5. Methoden

5.1 Intervention mit Multispezies Probiotikum

Das eingesetzte Multistrain-Probiotikum und das Placebo wird vom Institut “Allergosan” zur Verfügung gestellt. Es handelt sich hierbei um ein kommerziell erhältliches Nahrungsergänzungsmittel namens OMNi-BiOTiC® -SR welches 9 bakterielle Strains (*Bifidobacterium bifidum W23, Bifidobacterium lactis W51, Bifidobacterium lactis W52, Lactobacillus acidophilus W22, Lactobacillus casei W56, Lactobacillus paracasei W20, Lactobacillus plantarum W62, Lactobacillus salivarius W24, Lactococcus lactis W19*) mit zumindest 7.5 Billionen Mikroorganismen pro Portion enthält (=3g).

Das Placebopräparat wird die selbe Farbe, Konsistenz und den selben Geschmack haben wie das probiotische Produkt. Die Matrix des Produktes enthält Maisstärke, Maltodextrin, Inulin, Kaliumchlorid, Magnesiumsulfat, Fructooligosaccharide, Amylasen und Mangansulfat. PatientInnen und gesunde Kontrollen erhalten OMNi-Biotic SR nachdem dieses in Wasser aufgelöst und die Aktivierungszeit von 10 Minuten eingehalten wurde. Bei stationären PatientInnen wird das Studienpräprat täglich um 7:00 von Studienmitarbeitern vorbereitet und den stationären StudienteilnehmerInnen vor dem Frühstück und abends jeden Tag bis zur Entlassung gegeben. Nach Entlassung erfolgt die weitere Einnahme 2x täglich zu Hause selbstständig durch die PatientInnen. Das gesamte Studienteam ist verblindet.

5.2 Fragebögen

Um die Diagnose zu verifizieren wird bei depressiven PatientInnen welche stationär an der Univ. Klinik für Psychiatrie und psychotherapeutische Medizin aufgenommen wurden ein **M.I.N.I** durchgeführt. Die Schwere der depressiven Symptomatik wird mit der **Hamilton Scale for Depression (HAMD)** und dem **Beck Depression Inventory (BDI)** erfasst. Ergänzend wird die Schlafqualität mit dem **Pittsburgh Sleep Quality Inventory (PSQI)** erfasst. Die kognitive Reaktivität zu depressiver Stimmung wird mit dem **Leids-R Fragebogen** erfasst. Stress wird mit dem **Trierer Inventar zum chronischem Stress (TICS)** erfasst. Zusätzlich wird die **Perceived Stress Scale** zur Erhebung einer rezenten Stressbelastung erhoben. Das Bindungsverhalten erfassen wir mit der **Adult Attachment Scale**. Ergänzend erfassen wir folgende klinische und demographische Parameter: Größe, Gewicht, BMI, Geschlecht, Alter, Rauchverhalten, Medikation. Ernährungsanamnese mittels **Wiener Ernährungsprotokoll** (24-h-Recall), Bewegungsanamnese mittels **International Physical Acitivity Questionnaire**. Die Komplettierung dieser Fragebögen nimmt ca. 1-1,5 Stunden Zeit in Anspruch.

5.3 Herzratenvariabilität (HRV)

Zur Erfassung der Herzratenvariabilität wird der tragbare EKG Monitor "*eMotion Faros*" (http://ecg.biomation.com/faros.htm) eingesetzt. Das kleine und portable EKG-Gerät wird am Oberkörper der TeilnehmerInnen angebracht und zu den Testzeitpunkten für 24-h getragen. Das Gerät ist leicht handzuhaben und wird bereits erfolgreich in mehreren Bereichen wie Arbeitsmedizin, Kardiologie, Forschung, Physiotherapie, Sportberatung und Lebensstilberatung eingesetzt.

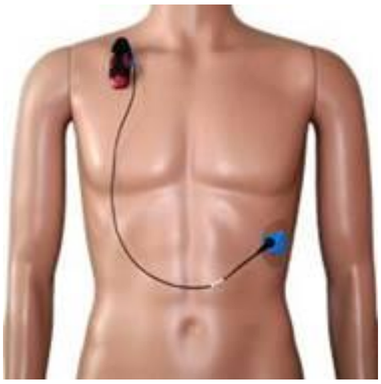

Abbildung 2: EKG-Gerät zur Erfassung der Herzratenvariabiltät. Technische Details::- ECG Sampling bis 250 Hz (adjustable); HRV: 1000 Hz sampling; 3D Acceleration (activity): sampling up to 25 Hz (adjustable).

Die HRV Analyse wird nach den Task-Force guidelines erfolgen (8). Die folgenden Parameter werden berechnet um die autonome Regulation zu erfassen: standard deviation of all normal-to-normal (NN) intervals (SDNN); log-transformed value of respiratory sinus arrhythmia (logRSA); the natural logarithm (ln) of high frequency (lnHF) and low frequency (lnLF) band; ln of total spectral power (lnTOT); LF/HF ratio, Puls Atem Quotient (PRQ); und Schlafdauer.

5.4 Mikrobiomanalyse

Ein Gramm Stuhl wird zu 3 Testzeitpunkten eingesammelt und unmittelbar in einem -80er Gefrierschrank für weitere Analysen gelagert. Die Auswertung des Mikrobioms erfolgt mit Illumina MiSeq nach bereits publiziertem Procedere (Klymiuk et al., 2017) [32]. FastQ files werden für die Datenanalyse verwendet. Quantitative Insights Into Microbial Ecology (QIIME II) wird auf dem galaxy server der MedUni Graz zur Auswertung des Darmmikrobioms verwendet (galaxy.medunigraz.at). Je nach finanziellen Mitteln soll zusätzlich das Metabolom (aus Stuhl)

5.5 Blutabnahme

Die Blutabnahme wird bei allen TeilnehmerInnen nüchtern am Morgen durchgeführt. Insgesamt werden max. 70ml Blut abgenommen. Nach der Blutabnahme wird das Plasma bei 4000r/min über 15 Minuten zentrifugiert und bei -20C für weitere Bestimmungen gelagert. C-reaktives Protein (CRP) und (IL)-6 werden am Institut für Labordiagnostik (Prof. Mangge, Doz. Meinitzer) mittels Cobas 6000 (Roche Diagnostics, Mannheim, Deutschland) analysiert.

Folgende Parameter werden außerdem bestimmt:

Routineparameter: Blutbild inklusive Differentialblutbild, Leberwerte, Nierenwerte, Lipide, CRP, IL-6, Glukosestoffwechsel
Biomarker: oxidative Stressparameter, Oxytocin, neuroinflammatorische Marker (z.B. Interleukine, TNF a, INF, Tryptophanstoffwechsel, IGF), Neurotrophine (z.B. BDNF), je nach verfügbarem Budget durch Förderungen werden die Proben sofort analysiert bzw. zwischenzeitlich gelagert.

5.6 Speichelprobe

Zum Einschlusszeitpunkt in die Studie sowie nach 1 und 4 Wochen soll, wenn genügend finanzielle Mittel lukriert werden können, Alpha-Amylase im Speichel als Stressmarker gemessen werden. Die Speichel-Amylase ist als Stressmarker bei depressiven Patient*innen erhöht (26). Studien konnten zeigen, dass Probiotika die Alpha-Amylase bei Sportler*innen oder chirurgischen Patient*innen beeinflussen konnten (27, 28) - ob Probiotika bei depressiven Patient*innen einen Einfluss auf die Speichelamylase nehmen können wurde noch nicht untersucht. Zur Bestimmung der Alpha-Amylase wird eine Speichelprobe benötigt. Die Gewinnung erfolgt non invasiv indem Speichel mittels Salivette (Sarstedt, Deutschland) von den Teilnehmenden selbst laut Testanleitung (nüchtern, vorher soll nicht Zähne geputzt oder Mundspüllösungen verwendet werden) gewonnen, dann zentrifugiert und bis zum Analysezeitpunkt tiefgefroren wird. Die Auswertung wird in Zusammenarbeit mit dem Otto Loewi Forschungszentrum, Pathophysiologie und Immunologie erfolgen.

6. Statistik

6.1 Randomisierung

Durch das Studiendesign ergeben sich 4 Gruppen:

- **Depressive PatientInnen Probiotika/Placebo**
- **Gesunde ProbandInnen: Probiotika/Placebo**

Die ProbandInnen werden mittels computergestützter **4-er Blockrandomisierung** (randomization.com) entweder der Probiotika oder der Placebogruppe zugeteilt.

6.1 Datenauswertung und Evaluation

Die gewonnen Daten werden mittels IBM SPSS, Version 27 ausgewertet. Es erfolgt eine deskriptive Datenbeschreibung (Mittelwert, Standardabweichung, Prozentrang). Korrelationen der Variablen werden je nach Verteilung mittels Pearson Korrelations-Koeffizient oder Spearman’s Korrelationskoeffizienten berechnet. Fehlerwahrscheinlichkeiten unter $p < 0.5$ werden als statistisch signifikant gesehen. Mikrobiom-Analysen werden in R (Version 3.6) und Rstudio (Version 1.2.1555)(R-doundation, Vienna, Austria), die Visualisierungen mit Hilfe ggplot2 Library durchgeführt.

Es wird sowohl eine Intention-To-Treat- als auch eine Per-Protocol-Analyse durchgeführt.

- **Intention-To-Treat-Analyse:**

Alle Einschlüsse (Valid Cases + Drop Outs). Es werden alle erhobenen Parameter analysiert.

- **Per-Protocol-Analyse:**

Alle Einschlüsse ohne Zutreffen eines Drop-Out-Kriteriums (alle Valid Cases). Es werden alle erhobenen Parameter analysiert. Die Per-Protocol-Analyse ist das vorrangige Auswertungskonzept.

Drop-out Kriterien:

Folgende Ereignisse/Situationen führen zu einer Einstufung des betroffenen Falls als Drop Out und somit zu dessen Nichtberücksichtigung für die Per-Protocol-Analyse:

- Schwerwiegende Projektplanverstöße
- (Mit Ausnahme des Prüfprodukts:) Einnahme / Konsumation von (relevanten Mengen an) Probiotika
- Fehleinschluss
- Entfall des follow-ups
- Nichtbereitstellbarkeit der für die Berechnung der Hauptzielparameter benötigten Daten
- Entblindung
- Zurückziehung der Einwilligung zur Studienteilnahme durch den Patienten/ die Patientin

6.2 Überlegungen zur Fallzahl

Dieses Projekt ist eine Pilotstudie. Nach unserem besten Wissen wurden noch nie probiotische Effekte auf die Vagusfunktion bei depressiven PatientInnen untersucht. Eine Power-Analyse wurde mit G*Power 3.1.(29) durchgeführt. In der Kategorie F-Tests wurde als Verfahren „Repeated Measures ANOVA within-between interaction" gewählt. Bei einer niedrigen Effektstärke von 0.2, Alpha 0.05 und 95% Power ergibt sich bei 4 Gruppen (Gesunde ProbandInnen: Probiotikum/Placebo; Depressive PatientInnen: Probiotikum/Placebo) eine Gesamtanzahl von 80 TeilnehmerInnen (20 TeilnehmerInnen pro Gruppe).

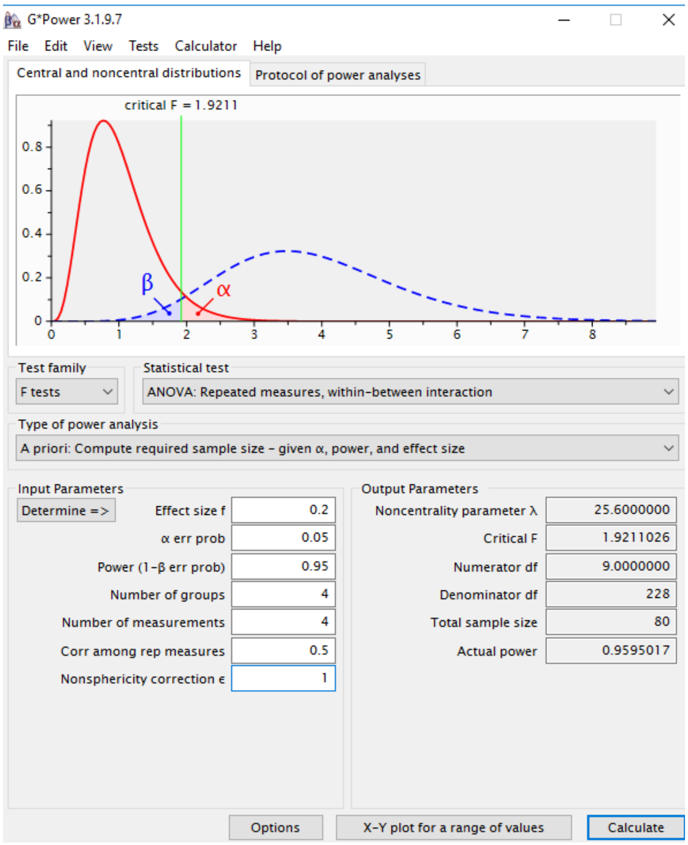

Abbildung 3: Fallzahlberechnung mit G*Power

6.3 Mikrobiomstatistik

Mikrobiomstatistik wird mittels R (v3.6) and Rstudio (1.2.1555) (R-foundation, Vienna, Austria) in Zusammenarbeit mit unserem Kooperationspartner Dr. Thomaz Bastiaanssen, APC Microbiome Institute Cork durchgeführt. Die Visualisierung der Daten wird mit der ggplot2 library vorgenommen. Um die Alpha Diversität zwischen der Interventions- und Placebogruppe zu vergleichen werden der Chao-1 Diversitätsindex, der Simpson-Index und der Shannon-Index

herangezogen. Die Diversitätsindices werden mittels iNEXT library [35] berechnet. CoDa wird aufgrund der kompositionellen Natur von Mirkobiomdaten verwendet (Gloor, et al., 2017). Beta-Diversität wird mit Principal Component Analysis (PCA) erfasst. Zur Korrektur für multiples Testen wird Storey's q-value post-hoc procedure mit einem q-value von 0.1 als cut-off (30) verwendet. Piphillin wird für funktionelle Mikrobiomanalysen herangezogen (prediction of metagenomic content)(31).

Referenzen

1. Klesse C, Berger M, Bermejo I, Bschor T, Gensichen J, Harfst T, et al. Evidenzbasierte Psychotherapie der Depression. Psychotherapeut. 2010;55(3):247-63.
2. Sansone RA, Sansone LA. Antidepressant adherence: are patients taking their medications? Innovations in clinical neuroscience. 2012;9(5-6):41.
3. Valles-Colomer M, Falony G, Darzi Y, Tigchelaar EF, Wang J, Tito RY, et al. The neuroactive potential of the human gut microbiota in quality of life and depression. 2019;4(4):623-32.
4. Sanada K, Nakajima S, Kurokawa S, Barceló-Soler A, Ikuse D, Hirata A, et al. Gut microbiota and major depressive disorder: A systematic review and meta-analysis. 2020;266:1-13.
5. Berthoud HR, Blackshaw LA, Brookes SJ, Grundy D. Neuroanatomy of extrinsic afferents supplying the gastrointestinal tract. Neurogastroenterol Motil. 2004;16 Suppl 1:28-33.
6. Egerod KL, Petersen N, Timshel PN, Rekling JC, Wang Y, Liu Q, et al. Profiling of G protein-coupled receptors in vagal afferents reveals novel gut-to-brain sensing mechanisms. Mol Metab. 2018;12:62-75.
7. Sgoifo A, Carnevali L, Pico Alfonso MdlA, Amore MJS. Autonomic dysfunction and heart rate variability in depression. 2015;18(3):343-52.
8. Bassett DJA, Psychiatry NZJo. A literature review of heart rate variability in depressive and bipolar disorders. 2016;50(6):511-9.
9. Matteoli G, Boeckxstaens GE. The vagal innervation of the gut and immune homeostasis. Gut. 2013;62(8):1214-22.
10. Jarczok MN, Kleber ME, Koenig J, Loerbroks A, Herr RM, Hoffmann K, et al. Investigating the associations of self-rated health: heart rate variability is more strongly associated than inflammatory and other frequently used biomarkers in a cross sectional occupational sample. 2015;10(2):e0117196.
11. Scott D, Happell B. The high prevalence of poor physical health and unhealthy lifestyle behaviours in individuals with severe mental illness. Issues Ment Health Nurs. 2011;32(9):589-97.
12. Teasdale SB, Ward PB, Samaras K, Firth J, Stubbs B, Tripodi E, et al. Dietary intake of people with severe mental illness: systematic review and meta-analysis. Br J Psychiatry. 2019;214(5):251-9.
13. Browning JS, Houseworth JH. Development of new symptoms following medical and surgical treatment for duodenal ulcer. Psychosom Med. 1953;15(4):328-36.
14. Whitlock FA. Some Psychiatric Consequences of Gastrectomy. Bmj-Brit Med J. 1961;1(523):1560-+.
15. Groves DA, Brown VJJN, Reviews B. Vagal nerve stimulation: a review of its

applications and potential mechanisms that mediate its clinical effects. 2005;29(3):493-500.
16. Mörkl S, Lackner S, Meinitzer A, Mangge H, Lehofer M, Halwachs B, et al. Gut microbiota, dietary intakes and intestinal permeability reflected by serum zonulin in women. 2018;57(8):2985-97.
17. Painold A, Mörkl S, Kashofer K, Halwachs B, Dalkner N, Bengesser S, et al. A step ahead: Exploring the gut microbiota in inpatients with bipolar disorder during a depressive episode. Bipolar Disord. 2019;21(1):40-9.
18. Reiter A, Bengesser SA, Hauschild A-C, Birkl-Töglhofer A-M, Fellendorf FT, Platzer M, et al. Interleukin-6 Gene Expression Changes after a 4-Week Intake of a Multispecies Probiotic in Major Depressive Disorder—Preliminary Results of the PROVIT Study. 2020;12(9):2575.
19. Gakis G, Mueller M, Hahn J, Glatzle J, Grundy D, Kreis MJAN. Neuronal activation in the nucleus of the solitary tract following jejunal lipopolysaccharide in the rat. 2009;148(1-2):63-8.
20. Goehler LE, Gaykema RP, Opitz N, Reddaway R, Badr N, Lyte M. Activation in vagal afferents and central autonomic pathways: early responses to intestinal infection with Campylobacter jejuni. Brain Behav Immun. 2005;19(4):334-44.
21. Bercik P, Park A, Sinclair D, Khoshdel A, Lu J, Huang X, et al. The anxiolytic effect of Bifidobacterium longum NCC3001 involves vagal pathways for gut–brain communication. Neurogastroenterology & Motility. 2011;23(12):1132-9.
22. Leistedt SJ, Linkowski P, Lanquart JP, Mietus J, Davis RB, Goldberger AL, et al. Decreased neuroautonomic complexity in men during an acute major depressive episode: analysis of heart rate dynamics. 2011;1(7):e27-e.
23. Kemp AH, Quintana DS, Gray MA, Felmingham KL, Brown K, Gatt JMJBp. Impact of depression and antidepressant treatment on heart rate variability: a review and meta-analysis. 2010;67(11):1067-74.
24. Mörkl S, Lackner S, Muller W, Gorkiewicz G, Kashofer K, Oberascher A, et al. Gut microbiota and body composition in anorexia nervosa inpatients in comparison to athletes, overweight, obese, and normal weight controls. Int J Eat Disord. 2017;50(12):1421-31.
25. Tunapong W, Apaijai N, Yasom S, Tanajak P, Wanchai K, Chunchai T, et al. Chronic treatment with prebiotics, probiotics and synbiotics attenuated cardiac dysfunction by improving cardiac mitochondrial dysfunction in male obese insulin-resistant rats. 2018;57(6):2091-104.
26. Bauduin SEEC, van Noorden MS, van der Werff SJA, de Leeuw M, van Hemert AM, van der Wee NJA, et al. Elevated salivary alpha-amylase levels at awakening in patients with depression. Psychoneuroendocrinology. 2018;97:69-77.
27. Kurdi M, Ramaswamy A, Kumar L, Choukimath S, Jangi A. Use of a non-invasive biomarker salivary alpha-amylase to assess the role of probiotics in sleep regulation and stress attenuation in surgical patients: A randomised double-blind clinical trial. Indian Journal of Anaesthesia. 2021;65(5):390-7.
28. Pumpa KL, McKune AJ, Harnett J. A novel role of probiotics in improving host defence of elite rugby union athlete: A double blind randomised controlled trial. J Sci Med Sport. 2019;22(8):876-81.
29. Faul F, Erdfelder E, Lang A-G, Buchner A. G* Power 3: A flexible statistical power analysis program for the social, behavioral, and biomedical sciences. Behavior research methods. 2007;39(2):175-91.
30. Storey JD, Tibshirani R. Statistical significance for genomewide studies. Proceedings of the National Academy of Sciences. 2003;100(16):9440-5.
31. Iwai S, Weinmaier T, Schmidt BL, Albertson DG, Poloso NJ, Dabbagh K, et al. Piphillin: improved prediction of metagenomic content by direct inference from human microbiomes. 2016;11(11):e0166104.
