# Supplementary material for: Do probiotics modulate dietary intake? Pilot data from a randomized controlled sub-study of the ProBioHRV clinical trial in patients with depression and healthy controls
Source: PLoS One. 2026 Jun 23;21(6):e0350801. doi: 10.1371/journal.pone.0350801 (PMC13289889; doi:10.1371/journal.pone.0350801)
Supplement: S6 File — (PDF) [file pone.0350801.s006.pdf]

Medical University of Graz
Ethics Committee

Auenbruggerplatz 2, A-8036 Graz
ethikkommission@medunigraz.at
Tel.: +43/ 316 / 385-13928, Fax: -14348

VOTE

Valid until 30 June 2022

**EC number:**	33-227 ex 20/21
**Study title:**	Pilot study: Probiotics and the gut-brain axis – Do probiotics interact with the vagal nerve?
**Examiner:**	Priv. Doz. DDr. Sabrina Mörkl<br>University Clinic for Psychiatry and Psychotherapeutic Medicine
**Sponsor:**	Medical University of Graz, University Clinic for Psychiatry and Psychotherapeutic Medicine<br>Priv. Doz. DDr. Sabrina Mörkl, 8036 Graz, Auenbruggerplatz 31
Contact person:	
CRO:	University Clinic for Psychiatry and Psychotherapeutic Medicine PD
Applicant: Contact person:	DDr. Sabrina Mörkl

The above-mentioned study was first discussed by the Ethics Committee at its meeting 05-20/21 on 8 February 2021.
The Ethics Committee has come to the following conclusion:

There are no objections to the study being conducted in its current form.

Members present and eligible to vote during the discussion were: See attached list dated 8 February 2021.

Committee members who were considered to have a conflict of interest in this agenda item and therefore did not participate in the decision-making and voting in accordance with the rules of procedure: none

Documents available for assessment:
Documents received on 18 January 2021, reviewed at meeting 05-20/21 on 8 February 2021

Cover Letter EK CoverLetter_V1_17012021 1	17 January 2021
Application form ECS	18 January 2021
Original minutes EK_Minutes_V1_17012021 1	17 January 2021
Informed consent form EK Patient information_V1 17012021 1	17 January 2021
Informed Consent Form EK_Control Information V1_17012021 1	17 January 2021
CV EK_CV_JolanaWagnerSkacel V1 19082020 1	19 August 2020
CV EK CV Pilz 05 2019 1	01 May 2019
CV CV Moerkl V1_17012021 1	17 January 2021
CV EK CV MelanieSchweinzer_V1 1	17 January 2021
CV EK_CV_Susanne Bengesser V1_19082020 1	19 August 2020
Miscellaneous: Hamilton questionnaire V1_17012021 1	17 January 2021
Miscellaneous: PSQI_V1 17012021 1	17 January 2021
Miscellaneous: TICS_V1 17012021 1	17 January 2021
Miscellaneous: LEIDS-R questionnaire_V1_17012021 1	17 January 2021
Miscellaneous: UKU_NW_Skala_V1_17012021 1	17 January 2021
Miscellaneous: BDI Test V1 17012021 1	17 January 2021

Medical University of Graz, Auenbruggerplatz 2, A-8036 Graz. www.medunigraz.at
Legal form: Legal entity under public law in accordance with UG 2002. Information: University newsletter UID: ATU 575 111 79. Bank details: Raiffeisen Landesbank Steiermark IBAN: AT4438000O0000049510, BIG: RZSTAT2G

Other: EK_ApplicationErlassProcessingFees_V1_17012021 1 — 17 January 2021
Miscellaneous: Vienna Nutrition Protocol_V1_17012021 1 — 17 January 2021
Miscellaneous: MINI 500_V1_17012021 1 — 17 January 2021

Documents received on 20 January 2021, reviewed at meeting 05-20/21 on 8 February 2021

ECS application form signed — 18 January 2021

Documents received on 18 February 2021 (to be reviewed in the next review)

Original protocol 1.1 — 18 February 2021
Informed consent form patient 1.1 Informed — 18 February 2021
consent form control 1.1 IPAQ questionnaires — 18 February 2021
undated
Adult Attachment Scale questionnaires, undated
Advertising material, flyer
Other: Statement on processing notification Other: Request for waiver — 18 February 2021
of processing fee — 18 February 2021
— 18 February 2021

Documents received on 4 March 2021 (to be reviewed in the next assessment)

Letter of authorisation — 4 March 2021

Documents received on 13 April 2021 (to be reviewed in the next assessment) Other: Support income
– Draft Allergosan/Med.Uni Graz

Documents received on 24 June 2021, reviewed in 'expedited **review'** on **30 June 2021**

Proof of payment — 9 June 2021

The Ethics Committee assumes – without legal obligation – that this does not constitute a clinical trial under the German Medicines Act (AMG) or Medical Devices Act (MPG).

This is a study conducted as part of a thesis.

The vote of the Ethics Committee does not in any way affect the sole responsibility of the investigator(s) for the proper conduct of the study in compliance with all relevant legal provisions and guidelines.

Furthermore, we would like to point out that the following must be reported to the committee immediately:

- Deviations from the protocol for safety reasons or protocol changes

- Changes that increase the risk to participants or significantly influence the conduct of the study

- Suspected unexpected serious adverse reactions (SUSARs) (AMG studies from 1 May 2004) or serious adverse events (SAEs) (other studies)

- Any information about other circumstances that may affect the safety of participants or the conduct of the study

Additional conditions: The measures prescribed by the authorities with regard to the COVID-19 pandemic must be observed. The investigator and the sponsor must ensure, within their respective spheres of influence and in compliance with any guidelines, that no resources required to combat the pandemic are tied up, that sufficient personnel are available and that participants are not exposed to any additional risk of infection as a result of their participation in the study.

This vote is valid for one year from the date of issue. If the study is to be conducted over a longer period, an interim report (report form) must be submitted in good time before the vote expires in order to obtain a possible extension.

Medical University of Graz, Auenbruggerplatz 2, A-8036 Graz. wu/w.medunigraz.at

Legal form: Legal entity under public law in accordance with UG 2002. Information – University newsletter. VAT ID: ATU 575 111 79. Bank details: Raiffeisen

Landesbank Steiermark IBAN: AT443800000000049510, BIC: RZSTAT2G

Graz, 30 June 2021

Univ. Prof. Josef Haas, Chairman

Dr Hans Peter Dimai Deputy Chairman

Please note Please quote the EK number in all correspondence or telephone enquiries relating to the project!

Medical University of Graz, Auenbruggerplatz 2, A-8036 Graz. wu/w.medunig at
Legal form: Legal entity under public law in accordance with UG 2002. Information: University Gazette. VAT ID: ATU 575 111 79. Bank details: Raiffeisen

Landesbank Steiermark IBAN: AT443800000000049510, BIC: RZSTAT2G

Medical University of G r a z
Ethics Committee

Auenbruggerplatz 2, A-8036 Graz
ethikkommission@medunigraz.at
Tel.: +43/ 316 / 385-13928, Fax: -14348

List of members entitled to vote or present

on 8 February 2021

Univ.Prof.DI Dr. Andrea Berghold
Univ.Prof. Dr. Hans Dimai Univ.Prof. Dr.
Thomas Griesbacher Univ.Prof.DI Dr. Josef
Haas
Iris Leitner-Englich
Dr. Regina Riedl
Univ.Prof.Dr. Peter J. Schick
Univ.Prof.Dr. Hermann Toplak PL
BSc. Esther Trampusch Ursula
Vennemann
Prof. Ursula Viktoria Wisiak Prof. Andreas
Zimmer Ing. Franz Deutschmann Prof.
Wolfgang Kröll Prof. Leopold Neuhold Prof.
Friedrich Reiterer Prof. Michael Speicher
Prof. Kurt Weber

Associated specialists

Associate Professor Jörg Lindenmann, PhD
Professor Freyja-Maria Smolle-Jüttner
Associate Professor PD Dr. Vanessa Stadlbauer-Köllner

Medical University of Graz, Auenbruggerplatz 2, A-8036 Graz. wu/w.medunigraz.at
Legal form: Legal entity under public law according to UG 2002 Information: University newsletter. VAT ID: ATU 575 111 79. Bank details: Raiffeisen

Landesbank Steiermark IBAN: AT443800000000049510, BIC: RZSTAT2G
