# Supplementary material for: Do probiotics modulate dietary intake? Pilot data from a randomized controlled sub-study of the ProBioHRV clinical trial in patients with depression and healthy controls
Source: PLoS One. 2026 Jun 23;21(6):e0350801. doi: 10.1371/journal.pone.0350801 (PMC13289889; doi:10.1371/journal.pone.0350801)
Supplement: S7 File — (PDF) [file pone.0350801.s007.pdf]

Medical University of Graz
Ethics Committee

Auenbruggerplatz 2, A-8036 Graz
ethikkommission@medunigraz.at
Tel.: +43/ 316 / 385-13928, Fax: -14348

SUBSEQUENT VOTE

Valid until 30 June 2023

**EC number:**	33-227 ex 20/21<br>1019-2021
**Study title:**	Pilot study: Probiotics and the gut-brain axis – Do probiotics interact with the vagal nerve?
**Investigator:**	Priv. Doz. DDr. Sabrina Mörkl<br>University Clinic for Psychiatry and Psychotherapeutic Medicine
**Sponsor:**	Medical University of Graz, University Clinic for Psychiatry and Psychotherapeutic Medicine
Contact person:	Priv. Doz. DDr. Sabrina Mörkl, 8036 Graz, Auenbruggerplatz 31
CRO:	
**Applicant:**	Medical University of Graz
Contact person:	PD DDr. Sabrina Mörkl, 8036 Graz, Auenbruggerplatz 31

The above-mentioned study was first discussed by the Ethics Committee at its meeting 05-20/21 on 8 February 2021.
The Ethics Committee has come to the following conclusion:

There are no objections to the study being conducted in its current form.

Members present and eligible to vote during the discussion were: See attached list dated 8 February 2021.

Committee members who were considered to have a conflict of interest in this agenda item and therefore did not participate in the decision-making and voting in accordance with the rules of procedure: none

Documents available for assessment:

Documents received on 18 January 2021, reviewed at meeting 05-20/21 on 8 February 2021

Cover Letter EK_CoverLetter_V1_17012021 1	17 January 2021
Application form ECS	18 January 2021
Original minutes EK_Protokoll_V1_17012021 1	17 January 2021
Informed consent form EK_Patient information_V1_17012021 1	17 January 2021
Informed Consent Form EK_Control Information_V1_17012021 1	17 January 2021
CV EK_CV_JolanaWagnerSkacel_V1_19082020 1	19 August 2020
CV EK_CV_Pilz_05_2019 1	01 May 2019
CV CV_Moerkl_V1_17012021 1	17 January 2021
CV EK_CV_MelanieSchweinzer_V1 1	17 January 2021
CV EK_CV_Susanne Bengesser_V1_19082020 1	19 August 2020
Miscellaneous: Hamilton questionnaire_V1_17012021 1	17 January 2021
Miscellaneous: PSQI_V1_17012021 1	17 January 2021
Miscellaneous: TICS_V1_17012021 1	17 January 2021
Miscellaneous: LEIDS-R_Questionnaire_V1_17012021 1	17 January 2021
Miscellaneous: UKU_NW_Skala_V1_17012021 1	17 January 2021

Medical University of Graz, Auenbruggerplatz 2, A-8036 Graz. www.medunigraz.at
Legal form: Legal entity under public law according to UG 2002. Information: University bulletin board UID: ATU 575 111 79. Bank details: Raiffeisen Landesbank Steiermark IBAN: AT44380000LE)00048510, BIC: RZSTAT2G

Other: BDI-Test_V1_17012021 1 17 January 2021

Miscellaneous: EK_Application for waiver of processing fees_V1_17012021 1 17 January

Miscellaneous: Vienna Nutrition Protocol II_V1_17012021 1 17 January

Miscellaneous: MINI 500_V1_17012021 1 17 January 2021

Documents received on 20 January 2021, reviewed at meeting 05-20/21 on 8 February 2021

ECS application form signed 18 January 2021

Documents received on 18 February 2021 (**to be reviewed** in the next **review)**

Original protocol 1.1

Informed consent form patient 1.1 Informed consent form control 1.1 IPAQ questionnaires undated

Adult Attachment Scale questionnaires, undated Advertising material, flyer

Other: Statement on processing notification Other: Request for waiver of processing fee

18 February 2021

18 February 2021

18 February 2021

Documents received on 4 March 2021 (to be reviewed in the next assessment) 18 February 2021

Letter of authorisation

Documents received on 13 April 2021 (to be reviewed in the next assessment) Other: Support income – Draft Allergosan/Med.Uni Graz 18 February 2021

Documents received on 24 June 2021, reviewed in 'expedited review' on 30 June 2021 Proof of payment 18 February 2021

Documents received on 4 July 2021, reviewed in an **expedited** review on 13 July 2021

4 March 2021

9 June 2021

Original minutes 1.2	03.07.2021
Informed consent form check 1.2	03.07.2021
Informed consent form patient 1.2	03.07.2021
Miscellaneous: EC reporting form – amendment	03.07.2021

Documents received on 30 August 2021, reviewed in an **expedited** review on 16 September 2021

Advertising material Advertising text for control subjects 1 27 August 2021

Advertising material Advertising text for patients 1 27

Documents received on 27 June 2022, reviewed in an expedited review on 8 July 2022 Interim report 27 June 2022

Date of initial vote: 30 June 2021

The Ethics Committee assumes – without legal obligation – that this is not a clinical trial according to the German Medicines Act (AMG) or Medical Devices Act (MPG).

This is a study conducted as part of a thesis.

The vote of the Ethics Committee does not in any way affect the sole responsibility of the investigator(s) for the proper conduct of the study in compliance with all relevant legal provisions and guidelines.

Furthermore, we would like to point out that the following must be reported to the committee immediately:

- Deviations from the protocol for safety reasons or protocol changes

- Changes that increase the risk to participants or significantly influence the conduct of the study

- Suspected unexpected serious adverse reactions (SUSARs) (AMG studies from 1 May 2004) or serious adverse events (SAEs) (other studies)

- Any information about other circumstances that may affect the safety of participants or the conduct of the study

Additional Requirements: The measures prescribed by the authorities with regard to the COVID-19 pandemic must be observed. The investigator and the sponsor must ensure, within their respective spheres of influence and in compliance with any guidelines, that no resources required to combat the pandemic are used.

MedlzInlsche Universiat Graz, Auenbruggerplatz 2, A-8036 Graz. www.medunigraz.at

Legal form: Juridical person under public law according to UG 2002 Information: University newsletter UID: ATU 575 111 79. Bank details: Raiffeisen Landesbank Steiermark IBAN: AT44380000C000049510, BIC: RZSTAT2G

Resources are available and sufficient staff are present, and participants are not exposed to any additional risk of infection through their participation in the study.

Graz, 8 July 2022

Univ.Prof.DI Dr.Josef Haas Chairman

Univ.Prof. Dr. Hans Peter Dimai
Deputy Chair

Please note Please quote the EK number in all correspondence or telephone enquiries relating to the project!

Medical University of Graz, Auenbruggerplatz 2, A-8036 Graz. www.medunlgraz.at
Legal form: Legal entity under public law gaTi UG 2002 Information: University bulletin board UID: ATU 575 111 79. Bank details: Raiffeisen Landesbank Steiermark IBAN: AT44380000€000049510, BIC: RZSTAT2G
