# Supplementary material for: Do probiotics modulate dietary intake? Pilot data from a randomized controlled sub-study of the ProBioHRV clinical trial in patients with depression and healthy controls
Source: PLoS One. 2026 Jun 23;21(6):e0350801. doi: 10.1371/journal.pone.0350801 (PMC13289889; doi:10.1371/journal.pone.0350801)
Supplement: S8 File — (PDF) [file pone.0350801.s008.pdf]

Medical University of Graz
Ethics Committee

Neue Stiftingtalstr. 6 - West, Q/04, A-8010 Graz
ethikkommission@medunigraz.at
Tel.: +43 / 316 / 385-13928

SUBSEQUENT VOTE

Valid until 30 June 2024

**EC number:**	33-227 ex 20/21
**Study title:**	1019-2021<br>Pilot study: Probiotics and the gut-brain axis – Do probiotics interact with the vagal nerve?
**Investigator:**	Priv. Doz. DDr. Sabrina Mörkl<br>University Clinic for Psychiatry and Psychotherapeutic Medicine
**Sponsor:**	Medical University of Graz, University Clinic for Psychiatry and Psychotherapeutic Medicine<br>Priv. Doz. DDr. Sabrina Mörkl, 8036 Graz, Auenbruggerplatz 31
Contact person:	
CRO:	Medical University of Graz
**Applicant:**	PD DDr. Sabrina Mörkl, 8036 Graz, Auenbruggerplatz 31
Contact person:	

The above-mentioned study was first discussed by the Ethics Committee at its meeting 05-20/21 on 8 February 2021.
The Ethics Committee has come to the following conclusion:

There are no objections to the study being conducted in its current form.

Members present and eligible to vote during the discussion were: See attached list dated 8 February 2021.

Committee members who were considered to have a conflict of interest in this agenda item and therefore did not participate in the decision-making and voting in accordance with the rules of procedure: none

Documents available for assessment:

Documents received on 18 January 2021, reviewed at meeting 05-20/21 on 8 February 2021

Cover Letter EK_CoverLetter_V1_17012021 1	17 January 2021
Application form ECS	18 January 2021
Original minutes EK_Protokoll_V1_17012021 1	17 January 2021
Informed consent form EK_Patient information_V1_17012021 1	17 January 2021
Informed Consent Form EK_Control Information_V1_17012021 1	17 January 2021
CV EK_CV_JolanaWagnerSkacel_V1_19082020 1	19 August 2020
CV EK_CV_Pilz 05_2019 1	01 May 2019
CV CV_Moerkl_V1_17012021 1	17 January 2021
CV EK_CV_MelanieSchweinzer_V1 1	17 January 2021
CV EK_CV_Susanne Bengesser_V1_19082020 1	19 August 2020
Miscellaneous: Hamilton questionnaire_V1_17012021 1	17 January 2021
Miscellaneous: PSQI_V1_17012021 1	17 January 2021
Miscellaneous: TICS_V1_17012021 1	17 January 2021
Miscellaneous: LEIDS-R_Questionnaire_V1_17012021 1	17 January 2021
Miscellaneous: UKU_NW_Skala_V1_17012021 1	17 January 2021

Medical University of Graz, Neue Stiftingtalstraße 6, **A-8010 Graz. www.medunigraz.at**
Legal form: Legal entity under public law in accordance with UG 2002 Information: University newsletter UID: ATU 575 111 79. Bank details: Raiffeisen Landesbank Steiermark IBAN: AT443800000000049510, 8IC: RZSTAT2G

Other: BDI-Test_V1_17012021 1 — 17 January 2021

Other: EK_Application for exemption from processing fees_V1_17012021 1 — 17 January

Miscellaneous: Vienna Nutrition Protocol II_V1_17012021 1 — 17

Miscellaneous: MINI 500_V1_17012021 1 — 17 January 2021

Documents received on 20 January 2021, reviewed at meeting 05-20/21 on 8 February 2021

ECS application form signed — 18 January 2021

Documents received on 18 February 2021 (to be reviewed in the next review)

Original protocol 1.1 — 18 February 2021

Informed consent form patient 1.1 — 18 February 2021

Informed consent form control 1.1 — 18 February 2021

IPAQ questionnaires undated

Adult Attachment Scale questionnaires, undated Advertising material, flyer — 18 February 2021

Other: Statement on processing notification Other: Request for waiver of processing fee — 18 February 2021

Documents received on 4 March 2021 (to be reviewed in the next assessment)

Letter of authorisation — 18 February 2021

Documents received on 13 April 2021 (to be reviewed in the next assessment)

Miscellaneous: Support income – Draft Allergosan/Med.Uni Graz — 4 March 2021

Documents received on 24 June 2021, reviewed in an expedited review on 30 June 2021

Proof of payment — 9 June 2021

Documents received on 4 July 2021, reviewed in 'expedited review' on 13 July 2021

Original minutes 1.2 — 03.07.2021

Informed consent form check 1.2 — 03.07.2021

Informed consent form patient 1.2 — 03.07.2021

Miscellaneous: EC Consent Form – Amendment — 03.07.2021

Documents received on 30 August 2021, reviewed in an expedited review on 16 September 2021

Advertising material Advertising text for control subjects 1 — 27 August 2021

Advertising material Advertising text for patients 1 — 27

Documents received on 27 June 2022, reviewed in an expedited review on 8 July 2022

Interim report — 27 June 2022

Documents received on 13 December 2022, reviewed in an expedited review on 3 January 2023

Advertising material Flyer undated Other: EK-Meldeformular — 13 December 2022

Documents received on 11 July 2023, reviewed in an expedited review on 19 July 2023

Interim report — 11 July 2023

Date of initial vote: 30 June 2021

The Ethics Committee assumes – without legal obligation – that this does not constitute a clinical trial under the German Medicines Act (AMG) or Medical Devices Act (MPG).

This is a study conducted as part of a thesis.

The vote of the Ethics Committee does not in any way affect the sole responsibility of the investigator(s) for the proper conduct of the study in compliance with all relevant legal provisions and guidelines.

Furthermore, we would like to point out that the following must be reported to the committee immediately:

- Deviations from the protocol for safety reasons or protocol changes

- Changes that increase the risk to participants or significantly influence the conduct of the study

- Suspected unexpected serious adverse reactions - SUSARs (AMG studies from 1 May 2004; Directive 2001/20 EC), SADEs (Regulations 74/2017 and 746/2107) or serious adverse events - SAEs (other studies)

Medical University of Graz, Neue Stiftingtaistraße 6, **A-8010 Graz. www.medunigraz.at**

Legal form: Legal entity under public law in accordance with UG 2002. Information: University newsletter UID: ATU 575 111 79. Bank details: Raiffeisen Landesbank Steiemark IBAN: AT443800000000049510, BIC: RZSTAT2G

- Any information about other circumstances that could compromise the safety of participants or the conduct of the study

Graz, 19 July 2023

Univ.Prof. Dr.DI Josef Haas
Chair

Univ.Prof.Dr.Hans Peter Dimai
Deputy Chair

Please note Please quote the EK number in all correspondence or telephone enquiries relating to the project!

Medical University of Graz, Neue Stiftfngtalstraße 6, A-8010 Graz. www.medunigraz.at
Legal form: Juridical person under public law in accordance with UG 2002. Information: **University** gazette **UID: ATU 575 111 79. Bank details: Raiffeisen**
Landesbank Steiermark IBAN: AT443800000000049510, BIC: RZSTAT2G
