# Supplementary material for: Do probiotics modulate dietary intake? Pilot data from a randomized controlled sub-study of the ProBioHRV clinical trial in patients with depression and healthy controls
Source: PLoS One. 2026 Jun 23;21(6):e0350801. doi: 10.1371/journal.pone.0350801 (PMC13289889; doi:10.1371/journal.pone.0350801)
Supplement: S9 File — (PDF) [file pone.0350801.s009.pdf]

Medizinische Universität Graz
Ethikkommission

Auenbruggerplatz 2, A-8036 Graz
ethikkommission@medunigraz.at
Tel.: +43 / 316 / 385-13928, Fax: -14348

VOTUM

gültig bis 30.06.2022

**EK-Nummer:**	33-227 ex 20/21
**Studientitel:**	Pilot-study: Probiotics and the gut brain axis- Do probiotics interact with the vagal nerve?
**Prüfer:**	Priv. Doz. DDr. Sabrina Mörkl<br>Universitätsklinik für Psychiatrie und psychotherapeutische Medizin
**Sponsor:**	Medizinische Universität Graz, Universitätsklinik für Psychiatrie und psychotherapeutische Medizin
Ansprechpartner:	Priv. Doz. DDr. Sabrina Mörkl, 8036 Graz, Auenbruggerplatz 31
**CRO:**	-
**Antragsteller:**	Univ. Klinik für Psychiatrie und psychotherapeutische Medizin
Ansprechpartner:	PD DDr. Sabrina Mörkl

Die o.a. Studie wurde von der Ethikkommission erstmals in der Sitzung 05-20/21 am 08.02.2021 behandelt.
Die Ethikkommission ist zu folgendem Schluss gekommen:

Es besteht kein Einwand gegen die Durchführung der Studie in der vorliegenden Form.

Stimmberechtigte bzw. anwesende Mitglieder bei der Behandlung waren: Siehe beiliegende Liste vom 08.02.2021.

Kommissionsmitglieder, die für diesen Tagesordnungspunkt als befangen anzusehen waren und daher gemäß Geschäftsordnung an der Entscheidungsfindung und Abstimmung nicht teilgenommen haben: keine

Zur Beurteilung vorliegende Dokumente:

Dokumente eingegangen am 18.01.2021, begutachtet in der Sitzung 05-20/21 am 08.02.2021

✓	Cover Letter EK_CoverLetter_V1_17012021 1	17.01.2021
✓	Antragsformular ECS	18.01.2021
	Originalprotokoll EK_Protokoll_V1_17012021 1	17.01.2021
	Informed Consent Form EK_PatientInnenaufklärung_V1_17012021 1	17.01.2021
	Informed Consent Form EK_Kontrollaufklärung_V1_17012021 1	17.01.2021
✓	CV EK_CV_JolanaWagnerSkacel_V1_19082020 1	19.08.2020
✓	CV EK_CV_Pilz_05_2019 1	01.05.2019
✓	CV CV_Moerkl_V1_17012021 1	17.01.2021
✓	CV EK_CV_MelanieSchweinzer_V1 1	17.01.2021
✓	CV EK_CV_SusanneBengesser_V1_19082020 1	19.08.2020
✓	Sonstiges: Hamilton Fragebogen_V1_17012021 1	17.01.2021
✓	Sonstiges: PSQI_V1_17012021 1	17.01.2021
✓	Sonstiges: TICS_V1_17012021 1	17.01.2021
✓	Sonstiges: LEIDS-R_Fragebogen_V1_17012021 1	17.01.2021
✓	Sonstiges: UKU_NW_Skala_V1_17012021 1	17.01.2021
✓	Sonstiges: BDI-Test_V1_17012021 1	17.01.2021

Medizinische Universität Graz, Auenbruggerplatz 2, A-8036 Graz. www.medunigraz.at
Rechtsform: Juristische Person öffentlichen Rechts gem. UG 2002. Information: Mitteilungsblatt der Universität. UID: ATU 575 111 79. Bankverbindung: Raiffeisen Landesbank Steiermark IBAN: AT443800000000049510, BIC: RZSTAT2G

Sonstiges: EK_AntragErlassBearbeitungsgebuehren_V1_17012021 1	17.01.2021
✓ Sonstiges: WienerErnährungsprotokoll_V1_17012021 1	17.01.2021
✓ Sonstiges: MINI 500_V1_17012021 1	17.01.2021
**Dokumente eingegangen am 20.01.2021, begutachtet in der Sitzung 05-20/21 am 08.02.2021**	
✓ Antragsformular ECS unterschrieben	18.01.2021
**Dokumente eingegangen am 18.02.2021 (in der nächsten Begutachtung mitbegutachtet)**	
✓ Originalprotokoll 1.1	18.02.2021
✓ Informed Consent Form Patient 1.1	18.02.2021
✓ Informed Consent Form Kontrolle 1.1	18.02.2021
✓ Fragebögen IPAQ undatiert	
✓ Fragebögen Adult Attachment Scale undtiert	
✓ Werbematerial Fleyer	18.02.2021
✓ Sonstiges: Stellungsnahme zur Bearbeitungsmitteilung	18.02.2021
✓ Sonstiges: Ansuchen Erlass Bearbeoitungsgebühr	18.02.2021
**Dokumente eingegangen am 04.03.2021 (in der nächsten Begutachtung mitbegutachtet)**	
✓ Letter of Authorization	04.03.2021
**Dokumente eingegangen am 13.04.2021 (in der nächsten Begutachtung mitbegutachtet)**	
✓ Sonstiges: Unterstützungsertrag - Draft Allergosan/Med.Uni Graz	
**Dokumente eingegangen am 24.06.2021, begutachtet im 'expedited Review' am 30.06.2021**	
✓ Zahlungsbeleg	09.06.2021

Die Ethikkommission geht - rechtlich unverbindlich - davon aus, dass es sich um keine klinische Prüfung nach AMG bzw. MPG handelt.

Es handelt sich um eine Studie im Rahmen einer Diplomarbeit.

Das Votum der Ethikkommission berührt in keiner Weise die alleinige Verantwortung der Prüferin / des Prüfers / der Prüfer für die ordnungsgemäße Durchführung der Studie unter Einhaltung aller einschlägiger gesetzlicher Bestimmungen und Richtlinien.

Weiters machen wir darauf aufmerksam, dass der Kommission unverzüglich zu melden sind:

- Abweichungen vom Protokoll aus Sicherheitsgründen oder Protokolländerungen

- Änderungen, die das Risiko der Teilnehmer/-innen erhöhen oder die Durchführung der Studie wesentlich beeinflussen

- Mutmaßliche unerwartete schwerwiegende Nebenwirkungen - SUSARs (AMG-Studien ab 1.5.2004) oder schwerwiegende unerwünschte Ereignisse - SAEs (andere Studien)

- Jegliche Information über sonstige Umstände, die die Sicherheit der Teilnehmer/-innen oder die Durchführung der Studie beeinträchtigen können

zusätzliche Auflagen: Die behördlich vorgeschriebenen Maßnahmen hinsichtlich der COVID-19 Pandemie müssen beachtet werden. Der Prüfer und der Sponsor müssen in ihrem jeweiligen Wirkungskreis unter allfälliger Beachtung von Leitlinien gewährleisten, dass keine zur Bekämpfung der Pandemie benötigten Ressourcen gebunden werden bzw. ausreichend Personal vorhanden ist und die TeilnehmerInnen durch ihre Studienteilnahme keiner zusätzlichen Infektionsgefahr ausgesetzt werden.

Dieses Votum gilt für ein Jahr ab dem Datum der Ausstellung. Bei längerer Studiendauer ist rechtzeitig vor Ablauf der Gültigkeit des Votums ein Zwischenbericht vorzulegen (Berichtsformular), um eine etwaige Verlängerung zu erlangen.

Medizinische Universität Graz, Auenbruggerplatz 2, A-8036 Graz. www.medunigraz.at
Rechtsform: Juristische Person öffentlichen Rechts gem. UG 2002. Information: Mitteilungsblatt der Universität. UID: ATU 575 111 79. Bankverbindung: Raiffeisen Landesbank Steiermark IBAN: AT443800000000049510, BIC: RZSTAT2G

Graz, 30. Juni 2021

Univ.Prof.DI Dr.Josef Haas
Vorsitzender

Univ.Prof.Dr.Hans Peter Dimai
Stv. Vorsitzender

Achtung: Bitte bei allen das Projekt betreffende Schreiben oder telefonischen Anfragen die EK-Nummer angeben!

Medizinische Universität Graz, Auenbruggerplatz 2, A-8036 Graz. www.medunigraz.at
Rechtsform: Juristische Person öffentlichen Rechts gem. UG 2002. Information: Mitteilungsblatt der Universität. UID: ATU 575 111 79. Bankverbindung: Raiffeisen Landesbank Steiermark IBAN: AT443800000000049510, BIC: RZSTAT2G

Medizinische Universität Graz
Ethikkommission

Auenbruggerplatz 2, A-8036 Graz
ethikkommission@medunigraz.at
Tel.: +43 / 316 / 385-13928, Fax: -14348

Liste der stimmberechtigten bzw. anwesenden Mitglieder

am 08. Februar 2021

Univ.Prof.DI Dr.Andrea Berghold
Univ.Prof.Dr. Hans Dimai
Univ.Prof.Dr.Thomas Griesbacher
Univ.Prof.DI Dr.Josef Haas
Iris Leitner-Englich
DI Dr.Regina Riedl
Univ.Prof.Dr.Peter J. Schick
Univ.Prof.Dr.Hermann Toplak
PL BSc. Esther Trampusch
Ursula Vennemann
Univ.Prof.Dr.Ursula Viktoria Wisiak
Univ.Prof.Dr.Andreas Zimmer
Ing.Franz Deutschmann
Univ.Prof.Dr.Wolfgang Kröll
Univ.Prof.Dr. Leopold Neuhold
Univ.Prof.Dr.Friedrich Reiterer
Univ.Prof.Dr. Michael Speicher
Univ.Prof.Dr.Kurt Weber

Beigezogene Fachärzte

Assoz. Prof. PD Dr.Jörg Lindenmann
Univ.-Prof. Dr.Freyja-Maria Smolle-Jüttner
Assoz.Prof.PD.Dr.Vanessa Stadlbauer-Köllner

Medizinische Universität Graz, Auenbruggerplatz 2, A-8036 Graz. www.medunigraz.at
Rechtsform: Juristische Person öffentlichen Rechts gem. UG 2002. Information: Mitteilungsblatt der Universität. UID: ATU 575 111 79. Bankverbindung: Raiffeisen Landesbank Steiermark IBAN: AT443800000000049510, BIC: RZSTAT2G
