# Supplementary material for: Do probiotics modulate dietary intake? Pilot data from a randomized controlled sub-study of the ProBioHRV clinical trial in patients with depression and healthy controls
Source: PLoS One. 2026 Jun 23;21(6):e0350801. doi: 10.1371/journal.pone.0350801 (PMC13289889; doi:10.1371/journal.pone.0350801)
Supplement: S11 File — (PDF) [file pone.0350801.s011.pdf]

Medizinische Universität Graz
Ethikkommission

Neue Stiftingtalstr. 6 - West, Q/04, A-8010 Graz
ethikkommission@medunigraz.at
Tel.: +43 / 316 / 385-13928

FOLGEVOTUM

gültig bis 30.06.2024

**EK-Nummer:**	33-227 ex 20/21<br>1019-2021
**Studientitel:**	Pilot-study: Probiotics and the gut brain axis- Do probiotics interact with the vagal nerve?
**Prüfer:**	Priv. Doz. DDr. Sabrina Mörkl<br>Universitätsklinik für Psychiatrie und psychotherapeutische Medizin
**Sponsor:**	Medizinische Universität Graz, Universitätsklinik für Psychiatrie und psychotherapeutische Medizin
Ansprechpartner:	Priv. Doz. DDr. Sabrina Mörkl, 8036 Graz, Auenbruggerplatz 31
**CRO:**	-
**Antragsteller:**	Medizinische Universität Graz
Ansprechpartner:	PD DDr. Sabrina Mörkl, 8036 Graz, Auenbruggerplatz 31

Die o.a. Studie wurde von der Ethikkommission erstmals in der Sitzung 05-20/21 am 08.02.2021 behandelt.
Die Ethikkommission ist zu folgendem Schluss gekommen:

Es besteht kein Einwand gegen die Durchführung der Studie in der vorliegenden Form.

Stimmberechtigte bzw. anwesende Mitglieder bei der Behandlung waren: Siehe beiliegende Liste vom 08.02.2021.

Kommissionsmitglieder, die für diesen Tagesordnungspunkt als befangen anzusehen waren und daher gemäß Geschäftsordnung an der Entscheidungsfindung und Abstimmung nicht teilgenommen haben: keine

Zur Beurteilung vorliegende Dokumente:

Dokumente eingegangen am 18.01.2021, begutachtet in der Sitzung 05-20/21 am 08.02.2021

✓	Cover Letter EK_CoverLetter_V1_17012021 1	17.01.2021
✓	Antragsformular ECS	18.01.2021
	Originalprotokoll EK_Protokoll_V1_17012021 1	17.01.2021
	Informed Consent Form EK_PatientInnenaufklärung_V1_17012021 1	17.01.2021
	Informed Consent Form EK_Kontrollaufklärung_V1_17012021 1	17.01.2021
✓	CV EK_CV_JolanaWagnerSkacel_V1_19082020 1	19.08.2020
✓	CV EK_CV_Pilz_05_2019 1	01.05.2019
✓	CV CV_Moerkl_V1_17012021 1	17.01.2021
✓	CV EK_CV_MelanieSchweinzer_V1 1	17.01.2021
✓	CV EK_CV_SusanneBengesser_V1_19082020 1	19.08.2020
✓	Sonstiges: Hamilton Fragebogen_V1_17012021 1	17.01.2021
✓	Sonstiges: PSQI_V1_17012021 1	17.01.2021
✓	Sonstiges: TICS_V1_17012021 1	17.01.2021
✓	Sonstiges: LEIDS-R_Fragebogen_V1_17012021 1	17.01.2021
✓	Sonstiges: UKU_NW_Skala_V1_17012021 1	17.01.2021

Medizinische Universität Graz, Neue Stiftingtalstraße 6, A-8010 Graz. www.medunigraz.at
Rechtsform: Juristische Person öffentlichen Rechts gem. UG 2002. Information: Mitteilungsblatt der Universität. UID: ATU 575 111 79. Bankverbindung: Raiffeisen Landesbank Steiermark IBAN: AT443800000000049510, BIC: RZSTAT2G

✓ Sonstiges: BDI-Test_V1_17012021 1	17.01.2021
Sonstiges: EK_AntragErlassBearbeitungsgebuehren_V1_17012021 1	17.01.2021
✓ Sonstiges: WienerErnährungsprotokoll_V1_17012021 1	17.01.2021
✓ Sonstiges: MINI 500_V1_17012021 1	17.01.2021
**Dokumente eingegangen am 20.01.2021, begutachtet in der Sitzung 05-20/21 am 08.02.2021**	
✓ Antragsformular ECS unterschrieben	18.01.2021
**Dokumente eingegangen am 18.02.2021 (in der nächsten Begutachtung mitbegutachtet)**	
✓ Originalprotokoll 1.1	18.02.2021
✓ Informed Consent Form Patient 1.1	18.02.2021
✓ Informed Consent Form Kontrolle 1.1	18.02.2021
✓ Fragebögen IPAQ undatiert	
✓ Fragebögen Adult Attachment Scale undtiert	
✓ Werbematerial Fleyer	18.02.2021
✓ Sonstiges: Stellungsnahme zur Bearbeitungsmitteilung	18.02.2021
✓ Sonstiges: Ansuchen Erlass Bearbeoitungsgebühr	18.02.2021
**Dokumente eingegangen am 04.03.2021 (in der nächsten Begutachtung mitbegutachtet)**	
✓ Letter of Authorization	04.03.2021
**Dokumente eingegangen am 13.04.2021 (in der nächsten Begutachtung mitbegutachtet)**	
✓ Sonstiges: Unterstützungsertrag - Draft Allergosan/Med.Uni Graz	
**Dokumente eingegangen am 24.06.2021, begutachtet im 'expedited Review' am 30.06.2021**	
✓ Zahlungsbeleg	09.06.2021
**Dokumente eingegangen am 04.07.2021, begutachtet im 'expedited Review' am 13.07.2021**	
✓ Originalprotokoll 1.2	03.07.2021
✓ Informed Consent Form Kontrolle 1.2	03.07.2021
✓ Informed Consent Form Patient 1.2	03.07.2021
✓ Sonstiges: EK-Meldeformular - Amendment	03.07.2021
**Dokumente eingegangen am 30.08.2021, begutachtet im 'expedited Review' am 16.09.2021**	
✓ Werbematerial Werbetext für Kontrollpersonen 1	27.08.2021
✓ Werbematerial Werbetext für PatientInnen 1	27.08.2021
**Dokumente eingegangen am 27.06.2022, begutachtet im 'expedited Review' am 08.07.2022**	
✓ Zwischenbericht	27.06.2022
**Dokumente eingegangen am 13.12.2022, begutachtet im 'expedited Review' am 03.01.2023**	
✓ Werbematerial Flyer undatiert	
✓ Sonstiges: EK-Meldeformular	13.12.2022
**Dokumente eingegangen am 11.07.2023, begutachtet im 'expedited Review' am 19.07.2023**	
✓ Zwischenbericht	11.07.2023

Datum Erstvotum: 30.06.2021

Die Ethikkommission geht - rechtlich unverbindlich - davon aus, dass es sich um keine klinische Prüfung nach AMG bzw. MPG handelt.

Es handelt sich um eine Studie im Rahmen einer Diplomarbeit.

Das Votum der Ethikkommission berührt in keiner Weise die alleinige Verantwortung der Prüferin / des Prüfers / der Prüfer für die ordnungsgemäße Durchführung der Studie unter Einhaltung aller einschlägiger gesetzlicher Bestimmungen und Richtlinien.

Weiters machen wir darauf aufmerksam, dass der Kommission unverzüglich zu melden sind:

- Abweichungen vom Protokoll aus Sicherheitsgründen oder Protokolländerungen

- Änderungen, die das Risiko der Teilnehmer/-innen erhöhen oder die Durchführung der Studie wesentlich beeinflussen

- Mutmaßliche unerwartete schwerwiegende Nebenwirkungen - SUSARs (AMG-Studien ab 1.5.2004; Directive 2001/20 EC), SADEs (Verordnung 74/2017 und 746/2107) oder schwerwiegende unerwünschte Ereignisse - SAEs (andere Studien)

- Jegliche Information über sonstige Umstände, die die Sicherheit der Teilnehmer/-innen oder die Durchführung der Studie beeinträchtigen können

Graz, 19. Juli 2023

Univ.Prof.DI Dr.Josef Haas
Vorsitzender

Univ.Prof.Dr.Hans Peter Dimai
Stv. Vorsitzender

Achtung: Bitte bei allen das Projekt betreffende Schreiben oder telefonischen Anfragen die EK-Nummer angeben!
